# DNA methylation landscapes of HIV controllers: an epigenome-wide association study

**DOI:** 10.1016/j.ebiom.2025.105999

**Published:** 2025-10-30

**Authors:** Manoj Kumar Gupta, Jéssica Cristina dos Santos, Victoria Ríos Vazquez, Suzanne D.E. Ruijten, Adriana Navas, Xun Jiang, Zhaoli Liu, Zhenhua Zhang, Javier Botey-Bataller, Onkar Singh, Nadira Vadaq, Albert L. Groenendijk, Wilhelm A.J.W. Vos, Marc J.T. Blaauw, Louise E. van Eekeren, Yang Li, Leo A.B. Joosten, Casper Rokx, Annelies Verbon, Vasiliki Matzaraki, Mihai G. Netea, Andre J.A.M. van der Ven, Cheng-Jian Xu

**Affiliations:** a Centre for Individualised Infection Medicine (CiiM), A Joint Venture Between the Helmholtz Centre for Infection Research (HZI) and the Hannover Medical School (MHH), Hannover, Germany; b TWINCORE, A Joint Venture Between the Helmholtz-Centre for Infection Research (HZI) and the Hannover Medical School (MHH), Hannover, Germany; c Department of Internal Medicine and Radboud Community for Infectious Diseases (RCI), Radboud University Medical Center, Nijmegen, the Netherlands; d Department of Internal Medicine and Department of Medical Microbiology and Infectious Diseases, ErasmusMC, Erasmus University, Rotterdam, the Netherlands; e Department of Medical Genetics, Iuliu Haţieganu University of Medicine and Pharmacy, Cluj-Napoca, Romania; f Department for Immunology and Metabolism, Life and Medical Sciences Institute (LIMES), University of Bonn, Bonn, Germany; g Department of Internal Medicine and Infectious Diseases, UMCU, Utrecht, the Netherlands

**Keywords:** HIV controllers, Epigenetics, DNA methylation, Epigenome-wide association study, MHC region

## Abstract

**Background:**

Spontaneous control of HIV infection without anti-retroviral therapy (ART) is a rare phenomenon observed in a small subset of people living with HIV (PWH), yet its underlying mechanisms remain poorly understood. Epigenetic modifications, particularly DNA methylation, may contribute to this unique phenotype.

**Methods:**

Here, we present an epigenome-wide association study (EWAS) analysing whole-blood DNA methylation profiles from the 2000HIV study, which includes HIV controllers (n = 111)-comprising elite controllers (ECs), viraemic controllers (VCs), transient controllers (TCs), and persistent controllers (PCs)- and virally suppressed PWH using ART (NHCs, n = 1667), from diverse ethnic backgrounds. The majority of participants (n = 1345) were of Western European descent, with additional representation from African and Asian populations.

**Findings:**

We identified and replicated three genome-wide significant CpG sites (cg04784635, cg13131185, and cg07189782) (FDR <0.05) with overlapping methylation patterns across various HIV controllers subtypes, including ECs. However, these methylation patterns exhibited population-specific differences, including African and Asian. Additionally, differential methylation region analysis revealed that cg17974398 in the major histocompatibility complex (MHC) region may mediate the effect of the genetic variant rs3131018, potentially contributing to HIV control.

**Interpretation:**

Our findings suggest that methylation near the MHC region may play an important role in HIV control and underscore the need for further investigation into population-specific epigenetic mechanisms.

**Funding:**

The work was conducted within the 2000HIV study, supported by 10.13039/100010877ViiV Healthcare.

**Research in context**

**Evidence before this study**

People living with HIV-1 (PWH) typically develop progressive immune deficiency unless treated with anti-retroviral therapy (ART). However, a small subset of PWH are able to control HIV infection either spontaneously or with minimal ART. Understanding the mechanisms underlying this natural control is essential for designing therapies aimed at achieving durable viral suppression in the absence of lifelong ART. Previous studies exploring these mechanisms have often been limited by small sample sizes and focused on single omics layers, such as gene expression or DNA methylation alone. Moreover, few studies have integrated functional immune profiling with epigenetic data. The 2000HIV cohort is the largest multi-omics cohort of PWH and uniquely enables the study of multiple omics layers, such as transcriptomics, epigenomics, and immunological data-generated from the same individuals. This resource provides an opportunity to investigate host regulatory mechanisms associated with HIV control on a systems level.

**Added value of this study:**

Our results showed significant differences between controllers and non-controllers, including epigenetic signature differences in genes involved with chromatin organisation and T-cell proliferation. We identified HIV control associated 3 CpG sites, namely, cg13131185, cg07189782, and cg04784635. Methylation near cg07189782, linked to *PRDX3*, was associated with reduced gene expression and protein levels, potentially aiding HIV control by limiting oxidative stress. This site also showed a previously unknown association with decreased immune activation markers and reduced CD8+ T cell cytotoxicity, suggesting a novel mechanism affecting HIV control. Additionally, methylation near *HLA-C* in the MHC region was linked to controller status, with evidence that genetic variants may influence HIV control via epigenetic modifications, highlighting complex molecular regulation. This finding was specific to people with HIV of Western European ancestry. However, methylation patterns near *TNF* and *LTB* in the MHC region on chromosome 6 were linked to controller status across all ancestries. These results highlight the important role of hypermethylation in the MHC region for HIV control and the need to identify ancestry-specific signatures to develop targeted therapies.

**Implications of all the available evidence**

Together with previous research, our findings highlight the important role of epigenetic regulation in HIV pathogenesis and immune control. Specifically, hypermethylation in the MHC region, especially near *TNF* and *LTB* genes, is consistently associated with HIV controller status across ancestries. These methylation changes likely modulate key immune pathways involved in viral suppression. Understanding how these epigenetic modifications influence cytotoxic T cell function and immune activation offers a promising avenue for developing targeted therapies. Future studies should focus on leveraging such ancestry-specific epigenetic signatures to enhance immune responses and HIV control.

## Introduction

People living with HIV-1 (PWH) mostly develop progressive immune deficiency unless anti-retroviral therapy (ART) is initiated. However, a small group of PWH spontaneously control HIV infection without ART (HIV controller). These individuals are categorised into two groups: elite controllers, who maintain undetectable plasma HIV RNA levels (<50 copies/mL) without therapy, and viraemic controllers, who sustain low but detectable viral loads (50–2000 copies/mL).^1^, ^2^, ^3^ Earlier studies, constrained by assay sensitivity, used higher thresholds to define control. For example, Lambotte et al. identified HIV controllers as those maintaining HIV RNA <400 copies/mL for over 10 years without ART-reflecting the detection limits of that period.^4^ Nevertheless, Olson et al. assessed several elite controller definitions using CASCADE data from 25,692 HIV seroconverters, and suggested elite controllers are best defined as individuals with either consecutive undetectable HIV-RNA measurements for ≥6 months or >90% of HIV-RNA values < 400 copies/mL over a period of ≥10 years, as these criteria are associated with a lower risk of disease progression.^5^ HIV control status is established after the acute phase of HIV infection and is maintained for a certain period of time afterwards. Depending on the long-term outcome of HIV controllers, this status may either be lost (transient controllers) or persist (persistent controllers) over time.^6^ For instance, earlier longitudinal studies show that a substantial proportion of HIV controller eventually lose control: approximately 17% after 5 years,^7^ 13% after 6 years,^8^ and up to 83% of elite controllers after a median of 17 years, losses 74.6% virological control, 66.1% immunological control, and only 16.9% remaining stable without progression.^9^ A thorough understanding of the mechanisms that play a role in spontaneous and sustainable HIV control may offer a mechanistic framework for developing therapies aimed at achieving prolonged viral suppression in the absence of ART.

Studies indicate that an interplay between viral and human host factors plays an important role in spontaneous HIV control.^10^ The immune system drives responses that promote the elimination of HIV-infected CD4+ T cells and promotes the integration of HIV proviruses into silenced heterochromatin regions of the genome.^11^^,^^12^ CD8+ T cells with increased functionality have been reported as crucial for spontaneous HIV control.^13^^,^^14^ Recent studies have also emphasised the role of innate immune cells, including myeloid cells such as dendritic cells and monocytes, as well as natural killer (NK) cells in spontaneous HIV control.^15^, ^16^, ^17^ Additionally, genetic studies show the importance of the immune system, as HLA class I alleles (HLA∗B57/27) are more prevalent in HIV controllers.^18^ In addition to genetics, differences in epigenetic regulation of gene expression through DNA methylation^19^ have also been proposed as important regulators of the immune system in the context of HIV infection. For example, Moron-Lopez and colleagues showed that the *TNF* expression in CD4^+^ T cells is regulated by DNA hypermethylation, and these DNA methylation patterns may vary between elite and non elite controller.^20^ Specifically, the DNA methylation profile of elite controllers was similar to that of uninfected individuals without HIV, with the majority of the differences in non-HIV controllers ascribed to the occurrence of uncontrolled HIV viraemia in PWH before the initiation of ART.^18^ Additionally, compared to uninfected individuals, viraemic PWH mostly had hypomethylated CpG sites, including immune genes, particularly those in the MHC region. These methylation patterns are partially restored once viraemia is suppressed by ART initiation.^21^ Hypermethylated regions have also been observed in regions where proviral DNA is integrated, promoting HIV transcriptional silencing and reducing the number of replicating viruses.^22^

This study investigates whole-blood DNA methylation profiles of HIV controllers and non-controllers using the Infinium MethylationEPIC array (EPIC array). We conducted an epigenome-wide association study (EWAS) on 99 HIV controllers and 1372 normal progressors on ART (non-HIV controllers, NHCs) in the discovery cohort, with an independent validation cohort of 12 HIV controllers and 295 non-controllers.^23^ DNA methylation associations with HIV control were examined at both individual CpG sites and across genomic regions. We also examined the consistency of the significantly differentially methylated sites (DMSs) across controller sub-groups, namely ART-naïve elite controllers (ECs), persistent controllers (PCs), and transient controllers (TCs). To understand the potential functional effect of those CpG sites and regions, we integrated these epigenetic markers with host genetics, gene expression, whole-blood immune cell phenotyping, and plasma proteomics.

## Methods

### Ethics

This study utilised datasets from the 2000 study cohorts, which have been comprehensively outlined, including the cohorts and experimental methodologies, by Vos et al.^23^ In brief, within the 2000HIV study, participants from the discovery cohort were recruited from three specialised Dutch HIV treatment centres, two university medical centres, and one large general hospital (Radboudumc Nijmegen, Erasmus MC Rotterdam, and OLVG Amsterdam). Participants from the validation cohort were recruited independently in a separate specialised HIV center of a large general hospital (Elisabeth-TweeSteden Ziekenhuis Tilburg). The 2000HIV study was approved by the Medical Ethical Review Committee Oost Nederland, Nijmegen, the Netherlands NL68056.091.81 and published at clinicaltrials.gov (2000HIV study–NTC03994835). All participants provided written informed consent prior to inclusion.

### Cohort

This recruitment strategy was deliberately designed to minimise site-specific biases and enhance the reproducibility of our findings. The criteria for inclusion of the participants were HIV-1 infection, age of 18 years or older, receiving ART for at least six months, and with a latest plasma HIV-1 RNA load of less than 200 copies/mL. On average, patients were on long-term treatment, 16.5 years for HIV controllers and 14 for normal progressors. Besides PWH on ART, we enrolled spontaneous HIV-1 controllers defined as i. non-viraemic (elite) controllers (EC) characterised by HIV-1 RNA <75 copies/mL for more than 12 months in the absence of cART with stable CD4 T cell counts (>500 cells/mm^3^), ii. viraemic controllers (VC) characterised by HIV-1 RNA <10.000 copies/mL for at least 5 years in the absence of cART with stable CD4 T cell counts, and iii. transient controllers (TC) exhibited plasma HIV-RNA levels exceeding 10,000 copies/mL after initially meeting the criteria for being classified as an EC or VC. In contrast to transient controllers, persistent HIV controllers or persistent EC are individuals who managed to maintain their HIV-RNA levels less than 10,000 or 75 copies/mL respectively. ART was initiated in a subset of HIV controllers during follow-up but always after first having fulfilled the criteria for being a HIV controller. Factors that contributed to ART initiation included transmission prevention, patient preference or adherence to new HIV treatment guidelines introduced in 2015, which recommended ART for all PLHIV regardless of viral load levels or CD4 counts. Sex was self-reported by participants at the time of enrolment. The criteria for exclusion were an absence of informed consent, insufficient communication because of language barriers or other problems, current pregnancy, detectable plasma viral hepatitis B DNA or hepatitis C RNA, and signs of any current acute infection. Blood samples were drawn after at least 4 h of fasting and transported overnight to Radboudumc or left overnight on the bench (Radboudumc samples), so all samples could be processed similarly the following day, including immune phenotyping, while whole blood and plasma samples were stored at −80 °C until further processing.

### Statistics

Statistical tests were conducted using R (version 4.2.0) (www.r-project.org). A p-value <0.05 after multiple testing using the Benjamini-Hochberg FDR was considered statistically significant. A norminal significance threshold of p-value <0.05 was applied for the region analysis replication, exploratory analysis to identify overall consistent patterns.

### DNA methylation measurements and quality control

The methylation study involved 1914 participants and DNA extraction from whole blood performed by the Radboudumc Genetics Department using the ChemagicStar system. The dataset was categorised based on participating centres, resulting in a discovery cohort (1592 participants) and a validation cohort (322 participants). Both cohorts underwent independent analysis, with DNA methylation values calculated from the original IDAT files using the minfi package^24^ in R (version 4.2.0). During the pre-processing phase, two samples with sex mismatches were excluded from the discovery cohort, and one low-quality sample from the validation cohort (with a call rate below 99%) was also removed. Additionally, probes (2756 in the discovery cohort and 2641 in the validation cohort) with methylation values missing in more than 10% of the samples (detected with p > 0.01), along with probes located on the sex chromosomes (totalling 19,627 probes), were excluded from further analysis. Considering that the majority of participants were of Western European descent (Table 1), we excluded probes containing single nucleotide polymorphisms (SNPs) at the target CpG sites, where the minor allele frequency (MAF) exceeded 5% in European populations. Furthermore, we removed probes mapping to multiple loci (n = 52,173), following the suggestion in,^25^ from both the discovery and validation cohorts. Subsequently, stratified quantile normalisation was applied to the methylation data.^26^ After quality control, 1590 samples and 793,762 probes in discovery, and 321 samples and 793,852 probes remained for downstream analysis. Subsequently, participants with missing information and using immunomodulatory drugs were also removed (Extended Data Fig. S1). In brief, the EWAS analysis included 99 HIV controllers and 1372 non-controllers in the discovery cohort, and 12 HIV controllers and 295 non-controllers in the validation cohort.

**Table 1:** Clinical and demographic characteristics of HIV controllers and non- HIV controllers (NHCs) across all ancestries.

Parameter	Group	Discovery			Validation		
		HIV controllers	NHCs	p	HIV controllers	NHCs	p
Total number		99	1372		12	295	
Sex	Male	73 (73.74%)	1181 (86.08%)	<0.05	8 (66.67%)	251 (85.08%)	NS
	Female	26 (26.26%)	191 (13.92%)		4 (33.33%)	44 (14.92%)	
Age (Years)	[Mean ± sd]	50.85 ± 11.71	51.59 ± 11.79	NS	52.25 ± 5.97 (3.91%)	53.13 ± 10.95 (3.91%)	NS
	Median [Min, Max]	51 [28, 77]	53 [19, 84]		51 [41, 64]	53 [20, 77]	
Weight Baseline (kg)	[Mean ± sd]	81.35 ± 14.64	80.44 ± 14.50	NS	86.56 ± 21.95 (3.91%)	82.44 ± 15.33 (3.91%)	NS
	Median [Min, Max]	80 [51.2, 125.7]	79 [47, 150]		83.65 [52.3, 125.8]	81 [49.3, 130]	
Height Baseline (cm)	[Mean ± sd]	176.56 ± 9.03	177.89 ± 8.88	NS	178.08 ± 9.78 (3.91%)	177.67 ± 9.36 (3.91%)	NS
	Median [Min, Max]	177 [154, 198]	178 [150, 207]		178.5 [164, 191]	178 [144, 198]	
BMI Baseline (kg/m^2^)	[Mean ± sd]	26.14 ± 4.77	25.40 ± 4.17	NS	27.33 ± 7.11 (3.91%)	26.08 ± 4.3 (3.91%)	NS
	Median [Min, Max]	25.39 [18.58, 46.21]	24.88 [15.48, 48.37]		25.99 [19.44, 43.53]	25.71 [17.63, 40.41]	
Ancestry	Asian	5 (5.05%)	67 (4.88%)	<0.05	0 (0%)	11 (3.73%)	NS
	African	13 (13.13%)	144 (10.5%)		2 (16.67%)	20 (6.78%)	
	Western European	62 (62.63%)	1018 (74.2%)		10 (83.33%)	255 (86.44%)	
	Hispanic	2 (2.02%)	42 (3.06%)		0 (0%)	1 (0.34%)	
	Mixed	17 (17.17%)	101 (7.36%)		0 (0%)	6 (2.03%)	
	Native American	0 (0%)	0 (0%)		0 (0%)	2 (0.68%)	
Smoking ever	No	32 (36.36%)	456 (35.74%)	NS	4 (36.36%)	95 (35.85%)	NS
	Yes	56 (63.64%)	820 (64.26%)		7 (63.64%)	170 (64.15%)	
Smoking currently	No	60 (68.18%)	871 (68.26%)	NS	7 (63.64%)	182 (68.68%)	NS
	Yes	28 (31.82%)	405 (31.74%)		4 (36.36%)	83 (31.32%)	
Number of pack years	[Mean ± sd]	20.79 ± 48.02	13.69 ± 20.03	NS	15.16 ± 16.8 (3.91%)	16.23 ± 22.15 (3.91%)	NS
	Median [Min, Max]	6 [0, 362.5]	5 [0, 164]		8 [0, 44]	3.5 [0, 119]	
Most likely risk behaviour	Blood products	0 (0%)	5 (0.36%)	<0.05	0 (0%)	0 (0%)	NS
	Vertical	0 (0%)	8 (0.58%)		0 (0%)	2 (0.68%)	
	Heterosexual	35 (35.35%)	262 (19.1%)		5 (41.67%)	77 (26.1%)	
	IV drug use	1 (1.01%)	17 (1.24%)		0 (0%)	1 (0.34%)	
	MSM	60 (60.61%)	1007 (73.4%)		7 (58.33%)	206 (69.83%)	
	Unknown	3 (3.03%)	73 (5.32%)		0 (0%)	9 (3.05%)	
HIV Duration (Years)	[Mean ± sd]	16.46 ± 7.63	14.11 ± 8.18	<0.05	16.26 ± 6.08 (3.91%)	11.71 ± 8.02 (3.91%)	<0.05
	Median [Min, Max]	15.08 [1.49, 39]	12.96 [0.52, 42]		17.7 [5.31, 24]	10.34 [0.51, 37]	
ART Duration (Years)	[Mean ± sd]	6.89 ± 3.94	12.02 ± 6.64	<0.05	7.52 ± 3.64 (3.91%)	10.29 ± 7.05 (3.91%)	<0.05
	Median [Min, Max]	6.42 [0.28, 17.88]	10.92 [0.42, 26.44]		8.13 [1.95, 13.4]	8.37 [0.47, 25.07]	
CD4 count (10ˆ9 cells/L)	[Mean ± sd]	0.88 ± 0.43	0.74 ± 0.29	<0.05	0.68 ± 0.23 (3.91%)	0.67 ± 0.28 (3.91%)	NS
	Median [Min, Max]	0.76 [0.35, 3.17]	0.71 [0.07, 2.2]		0.66 [0.39, 1.13]	0.66 [0.12, 1.66]	
Viral load in copies/mL (for detectable means)	[Mean ± sd]	316.25 ± 389.81	64.8 ± 60.22	<0.05	8.25 ± 28.58	3.75 ± 15.13	NS
	Median [Min, Max]	166 [27, 1110]	44 [21, 400]		99 [99, 99]	37 [21, 121]	
Protease inhibitors	No	96 (96.97%)	1231 (89.72%)	<0.05	11 (91.67%)	270 (91.53%)	NS
	Yes	3 (3.03%)	141 (10.28%)	<0.05	1 (8.33%)	25 (8.47%)	
CCR5 inhibitor	No	99 (100%)	1372 (100%)	NS	12 (100%)	294 (99.66%)	NS
	Yes	0 (0%)	0 (0%)		0 (0%)	1 (0.34%)	
Integrase inhibitors	No	57 (57.58%)	657 (47.89%)	NS	3 (25%)	100 (33.9%)	NS
	Yes	42 (42.42%)	715 (52.11%)		9 (75%)	195 (66.1%)	

**Notes:** Sd: Standard deviation; MSM: Men who have sex with men; P: P value represents the comparison between HIV controllers (HIC) and non-HIV controllers (NHC). NS: Not significant; Statistical Analysis: A t-test was used for the continuous variable. For the categorical variable, Fisher's exact test was applied for comparison between two groups.

### Association between covariates and DNA methylation

The first 30 principal components (PC) were obtained from the most variable 5000 CpGs using the prcomp function of the stats package in R. The relationship between continuous covariates and PC was analysed using robust linear regression (rlm), while categorical covariates were assessed using Analysis of Variance (ANOVA).

### Differential methylation analysis

Methylation β-values were computed as a percentage using the formula: β = M/(M + U + 100), where M and U represent the signal intensities of methylated and unmethylated states, respectively. These β-values were then transformed into M-values using the formula log 2 (β/(1—β)). The methylation values were used to estimate the proportions of six immune cell types using a modified version of Housman's approach through the ‘estimateCellCounts2’ function in FlowSorted.Blood.EPIC (version 2.6.0)^27^ package in R. To remove the influence of extreme outliers within the dataset,^28^ we applied trimming to the methylation data using (25th percentile—3∗IQR) and (75th percentile + 3∗IQR), where IQR = interquartile range. A generalised linear regression model was fitted. The HIV controller status was considered an outcome variable in the model, and correction was made for age, sex, methylation plate, and six estimated immune cell proportions (CD8T, CD4T, NK, Bcell, Mono, Neu). While analysing all ancestry, the first two principal components were corrected for both the discovery and validation cohort. CpGs were considered significant in the EWAS study if they have: (i) in the Discovery cohort, a p-value after multiple testing by False Discovery Rate (FDR) < 0.05; (ii) in the Validation cohort, consistent direction as observed in the discovery cohort and p-value after multiple testing by FDR <0.05; and (iii) in the meta-analysis, consistent direction as observed in the Discovery cohort with FDR <0.05, p-value after multiple testing by FDR <0.05 in the validation cohort, and an FDR (meta-analysis) < 0.05. The meta-analysis was performed using METAL,^29^ by using a fixed effect model, which assumes low heterogeneity across sites or regions. We also run a post hoc Cochran's Q test to confirm the consistent effects between discovery and validation cohorts.

Differentially methylated regions (DMRs) in the discovery cohort were identified using two methods. comb-p (version 0.32) and DMRcate (version 1.21.0). DMRs were considered significant based on the following criteria: a) A DMR must consist of more than one probe; b) Distance between adjacent probes in each DMR must be located within a 1000 base pair range; and c) Each DMR must have multiple-testing corrected p-values of less than 0.05 in both methods, with the Sidak correction applied for comb-p and the FDR correction used for DMRcate. To be conservative, we considered a DMR to be statistically significant only if it met this threshold in both methods. Overlapping DMRs identified as significant by both methods were considered significant in the discovery cohort. CpGs in each overlapping DMR were further subject to global test,^30^ separately, in the validation cohort. A global test was performed using summary statistics from the DMS analysis using the globaltest package (version 5.58.0).

The methylation patterns of validated CpGs in the HIV controllers were also compared between the non-controllers and various HIV controller subtypes (TC, EC, VC, and persistent controllers). The difference between the HIV controller subtypes and the non-controllers was assessed independently using Wilcoxon tests. Raw p-values were adjusted separately for multiple comparisons using the FDR correction method for each subtype. Associations with FDR values below 0.05 were considered statistically significant.

### PBMC transcriptome analysis

Bulk RNA sequencing of PBMCs was conducted utilising short-read sequencing with the latest Illumina technology (exceeding 30 million reads per sample). The sequencing reads were aligned to the human reference genome NCBI build 38 or the latest version available using the STAR aligner (version 2.7.3a). Gene expression levels were quantified with the HTSeqCount function from DESeq2 (version 1.38.1), employing the most recent Ensembl gene annotation (GENCODE release 27 and Ensembl version 90 based on GRCh38). The data normalisation and processing were carried out using the DESeq2 package. Subsequently, the gene coordinates were lifted down to hg19 by the liftOver package (version 1.26.0) of R to harmonise with the DNA methylation analysis.

### Plasma protein measurement

Plasma protein levels were evaluated using a multiplex proximity extension assay (PEA) provided by Olink® Proteomics AB (Uppsala, Sweden). The study utilised the Olink® Explore 3072 platform, which encompasses 3072 proteins organised into eight 384-plex panels targeting inflammatory, oncological, cardiometabolic, and neurological pathways. Protein levels were quantified as Normalised Protein Expression (NPX) values, following a quality control and normalisation process designed by Olink. NPX values are derived by subtracting the extension control and the plate values from Cq values. A correction factor is applied to shift the scale, and all values are reported in the Log2 scale. Batch effects across panels were corrected using bridging normalisation. Six proteins (IL-6, TNF, CXCL8, LMOD1, SCRIB, and IDO1) were measured in duplicate for quality control in each of the eight panels. High correlations between duplicates were observed, leading to the use of measurements from the inflammatory panel for further analysis. Proteins with a limit of detection (LOD) in 25% or more of the samples (n = 547) were excluded, leaving 2367 proteins for further analysis. Next, as part of the quality control process, principal component analysis (PCA) was performed using NPX values. Outliers were identified as samples with principal component one (PC1) and/or two (PC2) values exceeding four standard deviations (SD) from the mean. This step resulted in the exclusion of seven samples, leaving 1910 samples for analysis. Finally, DNA methylation-matched samples from individuals of Western European genetic ancestry were selected for this study.

### Phenotyping circulating immune cells

Whole blood samples were immunophenotyped using three flow cytometry panels, each containing 17-20 markers, with custom-made tubes holding dry antibodies from DURA Innovations Technology (Beckman Coulter). The panels are designed to determine the cellular proportions within the innate and adaptive immune system compartments, and to assess the expression of key markers associated with activation, exhaustion, and the evaluation of developmental stages in T- and B-cells. Cells were analysed on a twenty-one-colour, six-laser CytoFLEX LX system (Beckman Coulter), utilising Cytexpert software 2.3. Daily instrument calibration and quality control were performed using CytoFLEX Daily QC Fluorospheres (Beckman Coulter, catalogue #B53230), CytoFLEX Daily IR QC Fluorospheres beads (Beckman Coulter, catalogue #C06147), and SPHERO™ Rainbow calibration particles 6-peak (Spherotech Inc., catalogue #RCP-30-5A-6). Data analysis was conducted using Kaluza V2.1.2 and the Cytobank Platform V9.0 (Beckman Coulter).

### Genotyping, imputation, and quality control

DNA was extracted from whole blood samples collected from each participant. Genotyping for the 2000HIV multi-ethnic cohort was conducted using the Illumina Infinium Global Screening Array. To ensure high-quality data, raw genetic variants and samples underwent rigorous quality control using PLINK v1.90b. Variants with more than 5% missing genotypes and significant deviations from Hardy–Weinberg equilibrium (HWE; p-value <10^−6^) were excluded. HWE exact tests were stratified by ancestry. Samples with a call rate below 97.5% and heterozygosity rates exceeding three standard deviations from the mean for their reported ancestries group were removed. Variants passing quality filters were mapped from GRCh37 to GRCh38 coordinates using the UCSC liftOver tool (https://genome.sph.umich.edu/wiki/LiftOver#Binary_liftOver_tool). Additionally, alignment to the TOPMed Freeze 5 reference panel (https://bravo.sph.umich.edu/freeze5/hg38/) on genome build GRCh38 was performed using tools (https://www.well.ox.ac.uk/∼wrayner/tools/) from the McCarthy group. After quality control, 582,404 variants from 1864 participants were retained for imputation. These variants were uploaded to the TOPMed Imputation server and imputed using the TOPMed reference panel (version r2, GRCh38). Post-imputation, variants with low-quality scores (R2 < 0.3 or ER2 < 0.7) or minor allele frequency (MAF) below 1% were excluded using BCF tools, resulting in a dataset comprising 10,810,841 variants from 1864 participants.

During marker quality control, variants with a call rate below 95%, MAF below 1%, or significant HWE violations (p-value <1 × 10^−6)^ were excluded. Samples with call rates under 97.5%, heterozygosity rates beyond three standard deviations from the mean, or ancestries outliers identified via principal component analysis (PCA) were also removed. Ancestry outliers were defined as individuals whose genetic PC1 and/or PC2 values deviated by more than three standard deviations from the European reference population in the 1000 Genomes Project. Following imputation and filtering based on the same criteria, the discovery cohort retained 9,148,674 SNPs from 1003 individuals, while the validation cohort retained 9,130,602 SNPs from 257 participants.

### Function annotation analysis

The chromosomal position of each significant CpG probe associated with DMS and DMRs was annotated using IlluminaHumanMethylationEPICanno.ilm10b4.hg19^31^ package of R. CpG annotated genes were identified using the GREAT annotation (version 4.0.4) (hg19).^32^ Gene set enrichment analysis was performed using the Enrichr (version 3.2) package of R (Kuleshov et al., 2016). Gene ontology was performed for molecular function (MF), biological process (BP), and cellular component (CC), and pathway enrichment analysis was done with KEGG, Elsevier Pathway Collection (Els), or BioCarta database. Results with FDR <0.05 were considered significant.

### eQTMs analysis

In both discovery and validation cohorts, regression analysis was performed to assess the relationship between the methylation levels of validated CpGs and the expression levels of nearby transcripts (known as eQTMs) using the same covariates as in the EWAS.^33^^,^^34^ Subsequently, the p-value obtained was corrected for multiple testing using FDR. An association having the same direction in both cohorts and FDR <0.05 was considered significant and validated. Gene set enrichment analysis was performed using the Enrichr (version 3.2) package of R (Kuleshov et al., 2016).

### Associations with other omics

To assess the relationship between each significant CpGs probe and other omics, i.e., plasma proteins, cell percentage, cytokines, and genetics, raw values of other omics layers were initially normalised using the inverse rank transform. Association between each signal and other omics was performed using the same covariates and models as in the EWAS. Subsequently, the p-values obtained were corrected using FDR. The same analysis was performed in the validation cohort. Associations having the same direction as in the discovery cohort and FDR-values <0.05 were considered significant and validated.

### Mendelian randomisation and mediation analysis

The effect of a variant associated with DMS was tested for its effects in HIV controller using GWAS catalogue summary statistics^18^ and MethQTL.^35^ Mendelian randomisation (MR) was performed using the TwoSampleMR package in R, employing single-SNP MR with the default method (Wald ratio test), considering the p-value <0.05 significant. Further to cross-check, we also performed a mediation test using the mediation package (version 4.5.0), considering genotype as the causal factor, DNA methylation as a potential mediator, and HIV controller as the ultimate outcome.

### Visualisation

All the visual plots were created using either the ggplot 2 package (version 3.4.2) in R or the seaborn package (version 0.12.2) in Python (version 3.12.8), and gwascat (version 2.34.0) packages of R were utilised for visualising the nearby regions (±500 kb) (hg19) of each validated DMRs.

### Role of funders

The funding sources for this study had no role in the study design; collection, analysis, or interpretation of data; writing of the report; or the decision to submit the manuscript for publication.

## Results

### Whole blood DNA methylation profile of PWH from the 2000HIV study

**Fig. 1:**
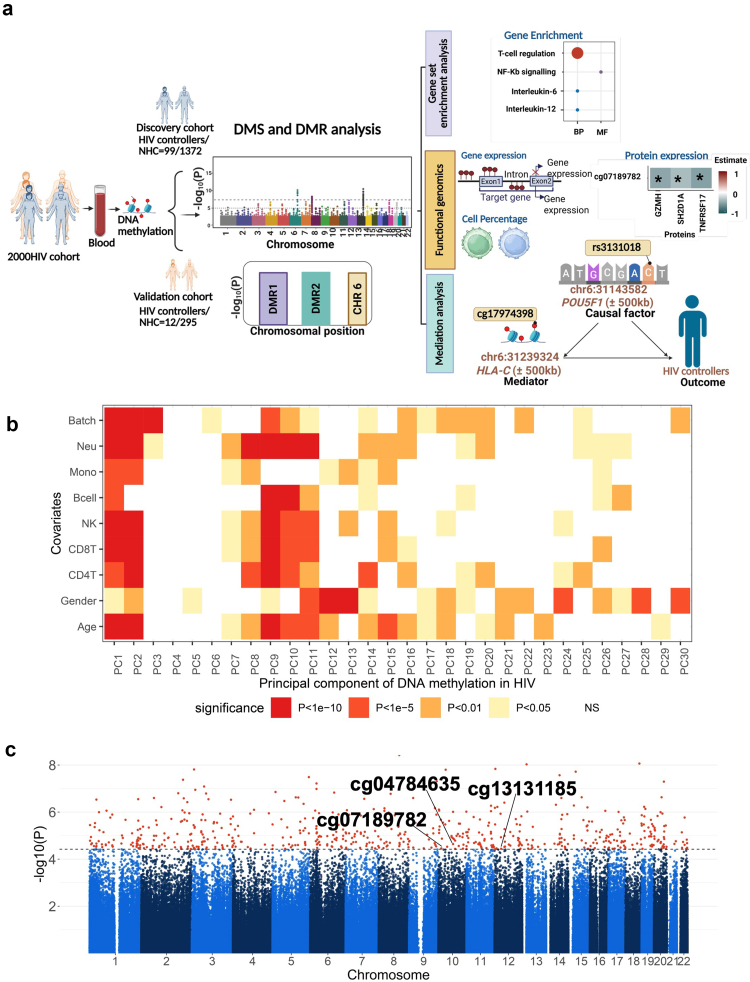
**Overview of study design and differential methylation analysis of HIV controllers. (a)** Methylation analysis of CpGs associated with HIV control was conducted in PWH from the 2000HIV cohort. The analysis was performed independently in the discovery (HIV controller = 99 and NHC = 1372) and validation cohorts (HIV controller = 12 and NHC = 295). Significant DMS were integrated with other omic layers, and downstream analyses, including gene set enrichment, functional genomics and mediation analysis, were performed **(b)** Heatmap showing the association of the first 30 PC with global DNA methylation variability in the discovery cohort, focussing on PWH of Western European ancestry. PC were calculated on the top 5000 most variable CpGs, with the top 30 PC collectively explaining 16.80% of the variance in DNA methylation data. The relationship between continuous covariates and PC was analysed using robust linear regression, while categorical covariates were assessed using Analysis of Variance. **(c)** Manhattan plot displaying the EWAS performed in the discovery cohort. The X-axis designates the chromosomal location of each CpG. Y axis designates the -Log_10_ (p-value) of each CpG. The dotted blue horizontal line represents the FDR significant threshold (FDR <0.05) of genome-wide significance. All 597 significant CpGs are marked in red. Red dots annotated are the 3 CpGs replicated in the validation cohort. EWAS was performed using a logistic regression model by adjusting for age, sex, methylation plate, and cell-type heterogeneity in DNA methylation. Fig. 1a was created with BioRender.com.

The study was conducted in PWH participating in the 2000HIV study, enrolled from four Dutch treatment centres, divided into a discovery and validation cohort.^23^ Whole blood DNA methylation profiles were available from 1778 subjects of various ancestries (Table 1 and Fig. 1a), with participants of Western European ancestry being the most prevalent. The analysis compared HIV controllers to virally suppressed, long-term ART-exposed non-controllers, NHCs. HIV controllers are divided in persistent controllers and transient controllers. The group of persistent HIV controllers comprises both viraemic controllers (VCs) and elite controllers. Viraemic controllers are defined as participants who maintain plasma HIV-RNA levels below 10,000 copies/mL and CD4 counts above 500 cells/μL for at least 5 years without ART. Elite controllers, representing approximately 25% of HIV controllers, are characterised by sustained plasma HIV-RNA levels below 75 copies/mL while remaining ART-naïve for at least 1 year. We defined transient controllers as PWH who initially met the criteria for spontaneous HIV control but later experienced loss of control, with plasma HIV-RNA levels exceeding 10,000 copies/mL. All TC from both the discovery and validation cohorts initiated ART, but ART was also given to VC in the discovery and validation cohorts, according to the new guidelines recommending ART for all PWH.^36^ HIV controllers from the discovery cohort differed from non-controllers in terms of sex, HIV duration, ancestry and CD4 counts (P_fisher.test_<0.05, Table 1). To further investigate differences in DNA methylation levels, DNA methylation was quantified using the Epic array, with pre-processing, normalisation, and stringent quality control.

We first studied participants of Western European ancestry, considering that DNA methylation profiles may vary among ancestries.^37^ The discovery cohort consisted of 1018 non-controllers and 62 HIV controllers, including 34 persistent controllers (of whom 15 were ECs) and 28 transient controllers. The validation cohort consisted of 10 HIV controllers, including two persistent controllers, eight transient controllers, and 255 non-controllers. When examining global DNA methylation variations, we observed that the top 30 PC explained 16.80% of the total variance. Among the tested covariates, age, sex, methylation plate, and estimated immune cell proportions showed the strongest associations with the top DNA methylation PC (Fig. 1b). In the validation cohort, the top 30 PC explained 25.26% of the variance, and the same set of variables remained the most strongly associated (Extended Data Fig. S2a).

### Epigenetic changes at CpGs involved in the regulation of inflammation, proliferation and activation of CD4^+^ T cells in HIV controllers

**Fig. 2:**
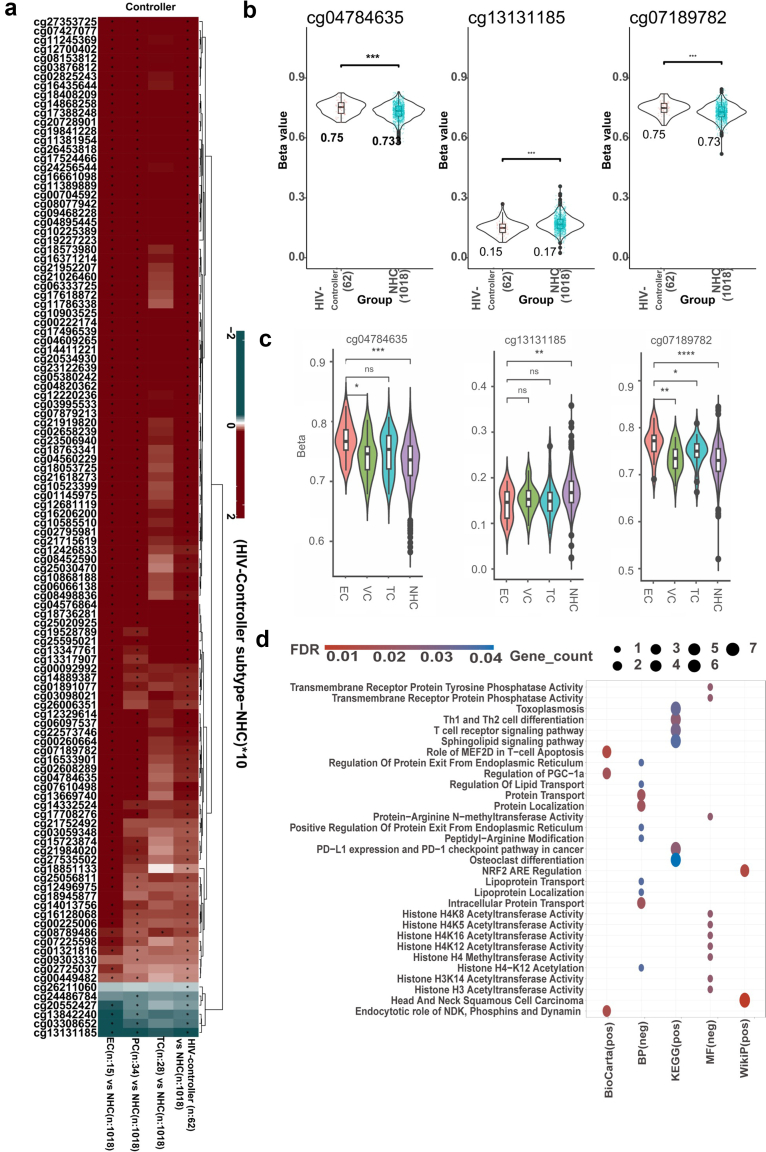
**Differentially methylation CpGs and their functional implications in HIV controllers.** (a) Heatmap showing the methylation pattern of the 112 nominally replicated (p-value <0.05) CpGs between HIV controllers and NHC of Western European origin. The methylation pattern of these CpGs was compared between the HIV controller (n = 62) subtypes (HIV, Transient controllers (TC, n = 28), persistent controllers (PCs, n = 34), Elite controllers (EC, n = 15) and non-controllers (n = 1018). Red designates the CpGs to experience hypermethylation in controllers than in non-controllers. Blue designates that the CpGs experience hypomethylation in HIV controllers as compared to NHC. Significance was tested using a Wilcoxon rank-sum test. Raw P-value were corrected using each subtype's False Discovery Rate (FDR). Associations having FDR <0.05 were considered significant and designated as∗. Violin plot showing the distribution of beta value in the three validated DMS (FDR <0.05) (b) between the same HIV controllers and NHC as well as (c) between the same different PWH groups type of the discovery cohort. Significance was tested using a Wilcoxon rank-sum test. Significance levels are indicated as follows: ns, not significant (p > 0.05); ∗, p ≤ 0.05; ∗∗, p ≤ 0.01; ∗∗∗, p ≤ 0.001; ∗∗∗∗, p ≤ 0.0001(d) Pathway enrichment analysis associated with 204 genes annotated with 112 DMS validated at nominal p-value (<0.05) from the EWAS. Pos and neg represent hypermethylation and hypomethylation, respectively. The top 10 significant pathways were plotted.

EWAS was performed using a logistic regression model to detect HIV controller-associated DMSs by adjusting for age, sex, methylation plate, and cell-type heterogeneity in DNA methylation. A Quantile–quantile (QQ) plot comparing observed versus expected -log_10_ (p-values) for all CpGs showed an inflation factor λ value of 1.29, indicating no strong evidence of model inflation (Extended Data Fig. S2b). We identified 597 DMSs in HIV controllers in the discovery cohort (FDR <0.05) (Fig. 1c and Table S1). Out of the 597, 112 DMS annotated 204 genes were confirmed in the validation cohort (nominal p-value <0.05, Fig. 2a, and Table S2) and three DMS reached statistical significance after multiple-testing correction (FDR <0.05) (Table 2 and Figs. 1c and 2b). These three most significant replicated DMSs were cg13131185 (chromosome 12), cg07189782 (chromosome 10) and cg04784635 (chromosome 10). Notably, cg07189782 and cg04784635 exhibited increased methylation in HIV controllers, specifically in ECs compared to non-controllers, while, for cg13131185, the methylation levels were decreased (Fig. 2b and c and Extended Data Fig. S3a). cg07189782 located within the *peroxiredoxin* (*PRDX) 3* gene (Extended Data Fig. S3b), which is a part of a group of peroxide scavengers known to play a role in the replication of viruses, including HIV. Moreover, peroxiredoxins are known for their role in the regulation of inflammation.^38^^,^^39^ Notably, the cg07189782 was previously reported to be associated with HIV infection.^40^

**Table 2:** Three validated significant DMS from EWAS analysis (HIV controllers vs NHCs) (sorted as per p value).

CpG	Chr.	Position (hg19)	Gene anno	MethylationΔ-disc	Eff-disc	P-disc	MethylationΔ-val	Eff-val	p-val	Eff-meta	P-meta	Cochran's Q statistics	Cochran's Q (p-value)
cg04784635	10	116,097,581	*AFAP1L2; VWA2*	0.02	2,68	1.16 × 10^−5^	0.03	3.54	2.42 × 10^−4^	2.93	1.44 × 10^−8^	0.57	0.45
cg13131185	12	113,914,611	*LHX5*	−0.02	−1,52	1.57 × 10^−5^	−0.04	−2.31	5.42 × 10^−6^	−1.77	8.31 × 10^−8^	1.64	0.20
cg07189782	10	120,935,712	*SFXN4; PRDX3*	0.02	2,48	2.42 × 10^−5^	0.03	2.97	9.30 × 10^−5^	2.67	9.97 × 10^−9^	0.25	0.62

CpG: CpG site identifier (Illumina probe ID); Chr: Chromosome number; Position: Genomic location; Gene Anno: Gene annotation; MethylationΔ-disc: Methylation difference (β value) in the discovery cohort between HIV controllers and NHCs; Eff-disc: Effect size from EWAS analysis in the discovery cohort; P-disc: p-value from EWAS analysis in the discovery cohort; Eff-val: Effect size from EWAS analysis in the replication cohort P-val: p-value from EWAS analysis in the replication cohort; Eff-meta: Effect size in the meta-analysis; P-meta: p-value in the meta-analysis; MethylationΔ-val: Methylation difference (β value) in the validation cohort between HIV controllers and NHCs; Cochran's Q statistics: Heterogeneity statistic across cohorts; Cochran's Q (p-value): p-value of the heterogeneity test.

Among the 112 nominally validated CpGs (p-value <0.05), two distinct patterns were observed when HIV controllers were compared to non-controllers: positive values indicate increased DNA methylation in HIV controllers (hypermethylation), while negative values indicate decreased methylation (hypomethylation) (Fig. 2a). Overall, HIV controllers showed a significantly greater (2.2 × 10^−16^, Proportion test) number of hypermethylated CpGs than hypomethylated ones in comparison with non-controllers. A distinct DNA methylation pattern in DMSs was also observed after stratifying the comparisons between HIV controller sub-groups and non-controllers. DNA hypermethylation was observed for the majority of replicated DMS in HIV controllers, such as elite controller, persistent control and transient controller. Although these DMSs did not reach statistical significance when comparing TC to NHC, TC still exhibited a trend toward hypermethylation at the majority of CpG sites. This suggests that across different groups of HIV controllers—ranging from ECs to TCs—an overlapping DNA methylation signature is observed.

Gene enrichment analysis of these 112 DMS annotated 204 genes showed significant enrichment (FDR <0.05) for signatures related to chromatin organisation, regulation of T cell activation and proliferation (*NFATC2, AKT3, IL12RB2, FYN* hyper-methylation in HIV controllers), and other pathways associated to T cell apoptosis such as *MEF2D* (hypermethylation in HIV controllers), known to sustain the activation of Foxp3^+^ T regulatory cells^41^ (Fig. 2d and Table S3).

### HIV controller-associated DMSs link to plasma GZMH, SH2D1A, TNFRSF17, and CD4+ T cells subtypes

**Fig. 3:**
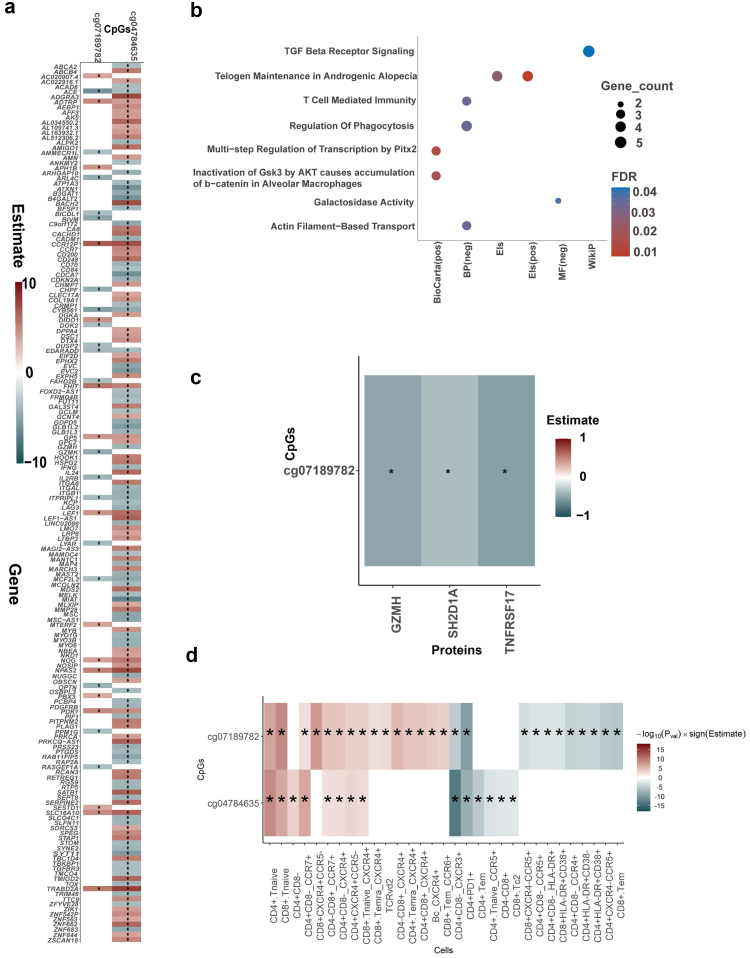
**HIV controllers -associated DMS in Western European negatively associated with plasma proteins.** (a) Heatmap showing the association between the validated DMS and gene expression in PBMCs, as well as (b) gene set enrichment analysis with the significant genes identified through eQTM. Pos and neg represent hypermethylation and hypomethylation in HIV controllers as compared to NHC, respectively. The association was also performed between the validated DMS and (c) plasma protein levels and (d) cell proportion. For plasma protein levels and cell proportion a robust linear regression was performed, adjusting for age, sex, methylation plate, and cell-type heterogeneity in DNA methylation∗ Depicts significant association (FDR <0.05).

To understand the potential regulatory function of those three validated DMSs (Table 2), we assessed their potential impact on gene expression through expression Quantitative Trait Methylation (eQTM) analysis. Results revealed two out of three DMSs showing significant associations (FDR <0.05) with 173 genes (Fig. 3a and Table S4). Both validated DMSs (cg07189782 and cg04784635) showed hypermethylation levels in HIV controllers compared to non-controllers. Specifically, hypermethylation at cg04784635 is negatively associated with the expression of genes, such as *CADM1, CD70* and *MYO1G,* which are involved in T-cell mediated pathways, as observed during the EWAS analysis (Fig. 3b and Table S5). These findings support the role of CpG methylation in the regulation of T-cell-mediated pathways in HIV controllers. We further explored the association between three validated CpGs and plasma protein concentrations and noticed that the methylation level of cg07189782 was negatively associated with circulating concentrations of three plasma proteins, namely Granzyme H (GZMH)*,* TNF receptor superfamily member (TNFRSF) 17*,* and SH2 domain-containing protein 1A (SH2D1A) (Fig. 3c, Table S6). GZMH, TNFRSF17 and SH2D1A are reported to be upregulated during HIV infection.^42^, ^43^, ^44^ Since DNA methylation may vary amongst cells,^45^ we subsequently performed the association between the three validated CpGs and the proportions of 355 immune cell types. The strongest association was observed between the negative correlation of cg04784635 with CXCR3+ expression in CD4^+^ T cells (P_adjust_ = 5.84 × 10^−11^) and the negative correlation between cg07189782 with PD-1 expression (P_adjust_ = 1.56 × 10^−7^) in CD4^+^ T cells (Fig. 3d and Table S7).

### DNA methylation changes in HIV controllers reveal critical role of MHC regulation in viral control

**Fig. 4:**
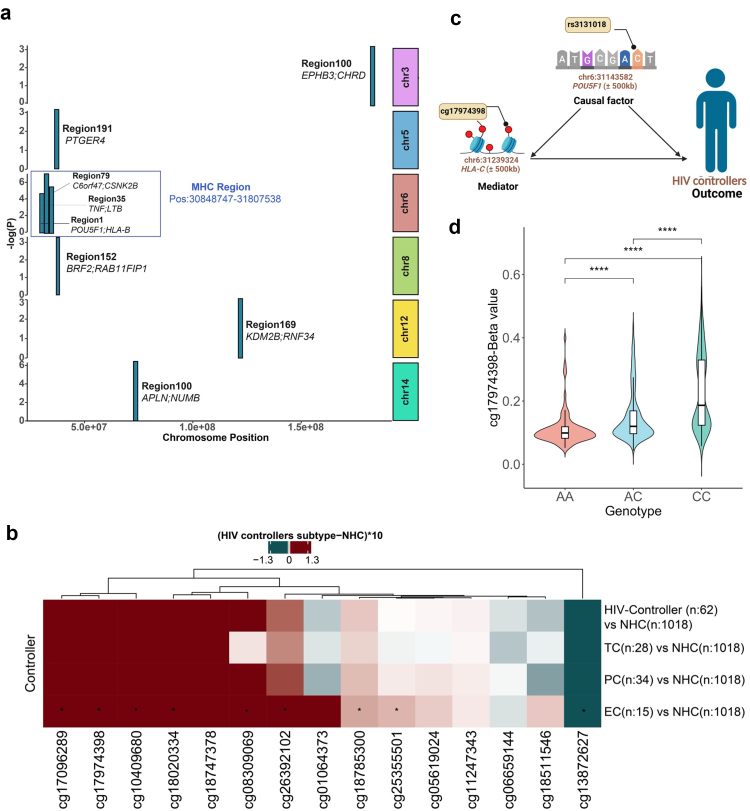
**DMR analysis of HIV controllers in Western European.** (a) Mantattan plot displaying eight validated DMRs (P-value <0.05) The X-axis represents the chromosomal location, and the y-axis represents the -log10 (p-value). DMRs in the blue box are three DMRs present in the MHC region on chromosome 6. (b) Heatmap showing the distribution of methylation Beta values for 15 CpG sites located in **region 1** on chromosome 6 (position: 31,237,824–31,240,651), near ***POU5F1*** and ***HLA-B***. Comparison was done between the HIV controller (n = 62), TC (n = 28), persistent controllers(PCs) (n = 34), EC (n = 15) and NHC (n = 1018). The x-axis represents CpG sites, while the y-axis represents the control group. The colour scale represents methylation differences, calculated as [HIV controllers subtype–NHC] × 10, with red indicating hypermethylation and blue indicating hypomethylation in HIV controllers as compared to NHC. Asterisks (∗) denote significant (p-value <0.05) differentially CpG sites. Hierarchical clustering is applied to group CpG sites based on similarity. (c) Mediation analysis revealed that the methylation signature of cg17974398 may mediate the function of genetic variant rs3131018-C near *POU5F1* in the MHC region and aid in HIV control. (d)The rs3131018-C allele is associated with higher methylation of cg17974398 compared to the rs3131018-A allele. AA (n = 97), AC (n = 468), and CC (n = 427). ∗∗∗∗ represent p ≤ 0.0001. For both (b) and (d), Significance was tested using a Wilcoxon rank-sum test. Fig. 4c was created with BioRender.com.

We next assessed the differentially methylated regions (DMRs) between HIV controllers and non-controllers from the discovery cohort using comb-P^46^ (Table S8) and DMRcate^47^ (Table 9). We identified 192 overlapping DMRs between the two methods (Table S10). Out of 192, eight DMRs annotated 15 genes (*POU5F1, HLA-B, EPHB3, CHRD, BRF2, RAB11FIP1, KDM2B, RNF34, PTGER4, TNF, LTB, PAPLN, NUMB, C6orf47,* and *CSNK2B*) were replicated with nominal levels of significance in the validation cohort (p-value <0.05) (Fig. 4a and Table S11).

Three DMRs (region 1, 35, and 79) are located in the Major Histocompatibility Complex (MHC) region on chromosome 6, whose association with HIV control, susceptibility and AIDS progression has been previously described^48^ (Fig. 4a, and Extended Data Figs. S4–S6). The identified DMRs revealed significant enrichment (FDR <0.05) for hits associated with NF-κB signalling pathways, including *TNF* and lymphotoxin beta (*LTB*) (Extended Data Fig. S7 and Table S12). Both *TNF* and *LTB* (region 35, Extended Data Fig. S5) are well-known mediators of inflammatory responses, and this region has previously been reported as being hypermethylated in CD4^+^ T cells from untreated viraemic PWH when compared to ECs and uninfected healthy controls.^20^

In addition to region 35, region 79 was comprised of CpGs nearby chromosome 6 open reading frame 47 (*C6orf47*) and casein kinase 2 beta (*CSNK2B*) genes, while region 1 consisted of CpGs nearby the transcription factor POU class 5 homoeobox 1 (*POU5F1*) and human leucocyte antigen (*HLA*)*-B* genes, all on chromosome 6.^49^^,^^50^ *POU5F1*, also known as Oct 3/4, has been reported as one of the transcription factors that are essential for maintaining pluripotency in haematopoietic stem cells,^51^ whereas *HLA-B* is well-known for its role in antigen presentation and allograft rejection responses. We observed that for most CpGs within these three regions, HIV controllers exhibited higher methylation levels compared to non-controllers. After stratifying the methylation values of region 1 across the different HIV controllers’ sub-phenotypes, we observed that the hypermethylation was significantly more pronounced in elite controllers, particularly at cg17974398, cg18785300, cg10409680, cg17096289, cg18020334, cg26392102, cg08309069, and cg25355501. Notably, cg13872627 showed hypomethylation in elite controllers compared to non-controllers, while methylation levels at the remaining CpGs in these regions did not differ between the two groups (Fig. 4b).

### SNP in the MHC contributes to HIV control through DNA methylation

The primary genetic determinants linked to spontaneous HIV control are specific SNPs within the MHC locus and classical MHC class I alleles^18^^,^^48^ (Fig. 4a, and Extended Data Figs. S4–S6). Our findings provided evidence that, in addition to genetics, epigenetic mechanisms may add an additional layer of regulation within the MHC locus, thereby contributing to HIV control. We further investigated the relationship between genetic variation and the observed DNA methylation changes in HIV controllers by performing single-SNP Mendelian randomisation (MR) using the GWAS and methQTL dataset from the PhenoScanner V2 database.^52^ A strong positive causal relationship (MR: P = 4 × 10^−16^) was identified between the rs3131018-C variant near the *POU5F1* gene and HIV control, primarily mediated through increased methylation at cg17974398 (Fig. 4c, Table S13). We then performed mediation analysis using genetic and methylation datasets of Western European ancestry. The presence of rs3131018-A is associated with decreased odds of HIV control (P_GWAS-Phenoscanner_ = 3.5 × 10^−4^; OR = 0.44). We confirmed the relationship between changes at cg17974398 and HIV control (p-value <0.05 and Table S14) by demonstrating that rs3131018-C is linked to higher cg17974398 methylation than rs3131018-A (Fig. 4d). Thus, these findings may suggest the mediation role of DNA methylation.

### Epi-genome-wide association study in HIV controllers across multiple ancestries

**Fig. 5:**
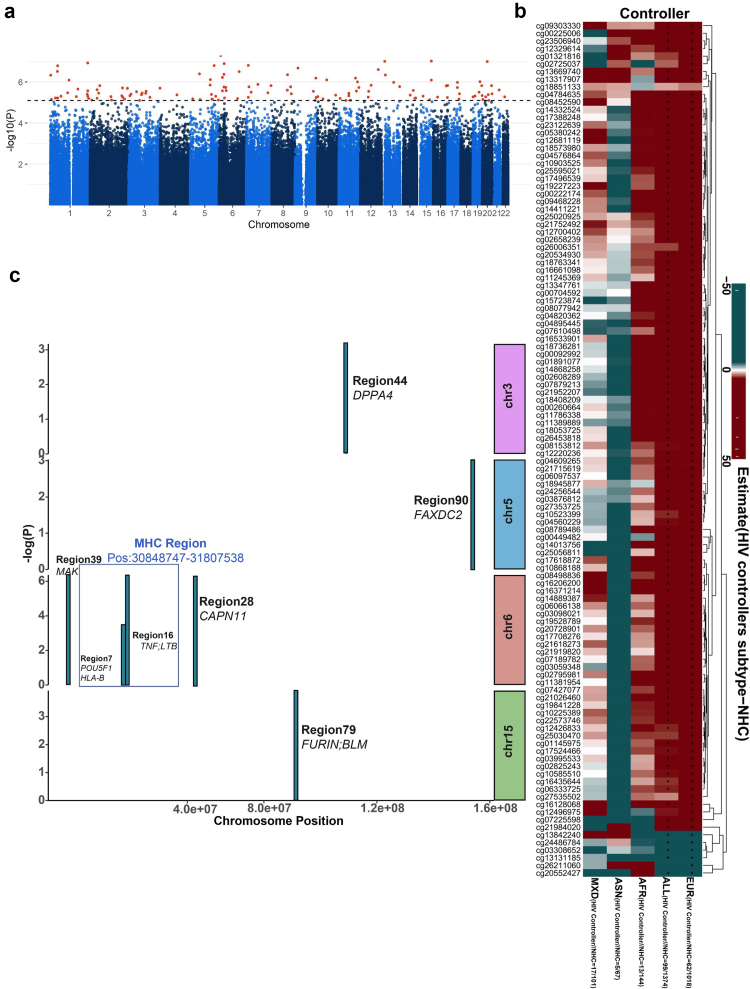
**HIV controller-associated DNA methylation pattern is population-specific.** (a) Manhattan plot showing -log_10_ (p-values) for CpGs associated with HIV controls in a multi-ancestry EWAS analysis. The X-axis designates the chromosomal location of each CpG. The Y-axis designates the -log_10_ (p-value) of each CpG. The dotted horizontal line represents the FDR significant threshold (FDR <0.05) of genome-wide significance. All 130 significant CpG sites in the discovery cohort are marked in red. EWAS was performed using a logistic regression model by adjusting for age, sex, methylation plate, and cell-type heterogeneity in DNA methylation as well as first two principal components of the genotype data. (b) Heatmap showing the methylation pattern in 112 nominally validated DMSs of Western European. EWAS was performed using the logistic regression model between HIV controllers (n = 62) vs. non-controllers (n = 1018) in European, all ancestries (ALL) (HIV controllers, n = 99; NHC, n = 1374), African (AFR) (HIV controllers, n = 13; NHC, n = 144), Asian (ASN) (HIV controllers, n = 5; NHC, n = 67), and Admixture (MXD) (HIV controllers, n = 17; NHC, n = 101). FDR correction is applied to these 112 CpGs per population. (c) 97 overlapping significant DMRs using two methods, namely comb-P and DMRcate, were detected in the discovery cohort. Out of 97, 7 DMRs annotated with 10 genes were nominally validated (p-value <0.05) in the replication cohort. The X-axis represents the chromosomal location, and the Y-axis represents the-log_10_ (p-value). DMRs in the blue box are 2 DMRs present in the MHC region.

Next, we conducted an epi-genome-wide association study of HIV controllers in all ancestries. To account for the heterogeneity of multi-ancestry populations, we included the first two principal components of the genotype data in the EWAS model. In the discovery cohort, 130 significant DMS (FDR <0.05) were detected (Fig. 5a and Table S15). The QQ plot showed no strong inflation of the model (λ = 1.28) (Extended Data Fig. S8). Of 130, no DMS could be validated in the validation cohort (FDR <0.05).

Further, to understand whether genetic ancestry may affect epigenetic patterns linked with HIV control, we analysed the methylation patterns of 112 nominally validated (p-value <0.05) DMS identified in the Western European population across multiple ancestries. These patterns were compared between HIV controllers and non-controllers across African, Asian, and mixed-ancestry populations. Distinct clustering was observed in the non-European populations compared to the Western European population (Fig. 5b). This finding suggests that the methylation patterns of the 112 nominally validated CpGs are population-specific, with particularly noticeable differences in the Asian population.

During the DMR analysis, we detected 97 overlapping significant DMRs via comb-p and DMRcate (Tables S16–S19). 7 DMRs annotated with 10 genes (*TNF, LTB*, *POU5F1, HLA-B, FURIN, BLM, CAPN11, MAK, DPPA4,* and *FAXDC2)* can be validated with nominal levels of significance (p-value <0.05) (Fig. 5c and Table S19). Amongst these seven DMRs, two are present in the MHC region (Fig. 5c and Extended Data Figs. S9 and S10). A comparison between validated DMRs in Western and across all ancestries reveals intriguing findings. DMRs containing *TNF* & *LTB* are identical between Western Europeans (Extended Fig. S4) and all ancestries (Extended Fig. S10). These findings suggest that while HIV controller-associated methylation patterns are population-specific, methylation patterns of gene regions nearby *TNF, LTB,* in the MHC region are common to all ancestries.

## Discussion

The present EWAS, which compared DNA methylation of spontaneous HIV controllers and virally suppressed PWH using ART, resulted in a rich data resource with DNA methylation signatures associated with HIV control in subjects of Western European ancestry. HIV is known to change the DNA methylation landscape,^21^ and we hypothesised that these changes would be smaller in controllers and more extensive in non-controllers, even after ART, as some epigenetic memory persists.^53^ Our results indeed showed significant differences between controllers and non-controllers, including epigenetic signature differences in genes involved with chromatin organisation and T-cell proliferation. This finding was limited to PWH of Western European ancestry. Notably, we found that methylation patterns in the MHC region on chromosome 6 nearby *TNF* and *LTB* were associated with controller status and were common across all ancestries.

Participants of Western European ancestry from the 2000HIV study^23^ were investigated to identify methylation signatures in HIV controllers and non-controllers using ART. Three groups of HIV controllers were selected, including ART-naïve elite controllers, viraemic controllers (of whom some were using ART because of changing guidelines, and not because of control loss), and finally, transient controllers that lost control status and were all on ART. It is pertinent to note that in several studies,^54^, ^55^, ^56^, ^57^, ^58^, ^59^ normal progressor PWH are commonly defined by a viral load cutoff >10,000 copies/mL and viraemic controllers by a cutoff of <2000 copies/mL. For the 2000HIV-study however, a threshold of 10,000 copies/mL was chosen for viraemic controllers for three main reasons. First, we aimed to study the full spectrum of HIV controllers and to evaluate viral loads in HIV controllers either by stratification or as a continuous measurement. Our threshold allowed us to investigate whether biological correlates exist that distinguish the threshold of 1000 or 2000 from that of 10,000 copies/mL. Second, literature on clinical outcomes shows that PWH with <10,000 copies/mL have improved survival and less progression to AIDS than PWH with >10,000 copies/mL. Concerning the benefit of immediate ART, the START landmark study showed that this benefit did not persist to PWH with <5000 copies/mL or <3000 copies/mL,^57^ both being above the typical viraemic controller cutoff of <2000 copies/mL. Thus, a higher threshold in the present study increases the number of controllers and potentially the statistical power.

In agreement with previous studies,^60^^,^^61^ the spontaneous controllers of our cohort are enriched for HLA-B:57 and HLA-B:27.^62^ Females represented 9.8% of the total population but 19.4% of HIV controllers in the Western European (Table S20). A Fisher's Exact Test confirmed that this difference in sex distribution is statistically significant (p-value <0.05). While the European HIV epidemic is predominantly male, this enrichment of females among controllers^63^ may reflect known sex-related differences in immune response and viral load dynamics. It is pertinent to note that age and sex both influenced DNA methylation patterns in the discovery and validation cohorts. However, due to the limited sample size in the validation cohort, we adjusted only for age, which had the strongest effect, to minimise overfitting while still accounting for a key source of variability. Notably, an overall overlapping DNA (hyper)methylation landscape was seen across the three groups of HIV controllers, indicating a possible protective epigenetic memory, in contrast to non-controllers. Our cross-sectional design does not allow the analysis of whether this signature changed during loss of control and was regained after initiation of ART or whether this protective signature was (temporarily) lost. Other studies comparing HIV controllers and non-controllers showed that differences in methylation of anti-viral genes are also related to differences in gene expression,^64^ while another study demonstrated DNA hypermethylation patterns closer to Transcription Start Sites are associated with the presence of latently infected cells in HIV controller.^65^

Using an independent discovery and validation cohort, three DMSs, namely, cg13131185, cg07189782, and cg04784635, were validated at an FDR <0.05. The three CpG sites together explain an 0.08% of the variance in viral load, which may be due to the complex and multifactorial nature of epigenetic regulation and the rarity of HIV infection controllers. Using the multi-omics data available in the 2000HIV cohort, we further explored how the methylation signatures in the three validated DMS are associated with other omics layers, namely, PBMC transcriptome data, plasma protein expression levels, and phenotypes of circulating immune cells. Results indicated that hypermethylation near the cg07189782 annotated near *PRDX3* could negatively affect gene expression and plasma concentration of this protein, which may facilitate HIV control by limiting oxidative stress and preserving mitochondrial function, potentially reducing HIV-induced immune activation and cellular damage.^39^

Interestingly, cg07189782 showed a negative association with the expression of PD-1, HLA-DR, and CD38 in CD4+ T cells. Similarly, cg04784635 was negatively associated with the expression of PD-1, Tnaive-CCR5, and CXCR3+ in CD4+ T cells. These markers are known to be associated with HIV infection progression.^66^, ^67^, ^68^ To the best of our knowledge, we also observed a previously unreported association between cg07189782 and reduced CD8 cytotoxicity, suggesting that cg07189782 may contribute to impaired CD8 function and potentially impact HIV control. Also, it is pertinent to note that while cg04784635 showed strong associations with gene expression and immune cell phenotypes, it did not correlate with protein levels. This disconnect underscores the complexity of molecular regulation beyond transcription and highlights the challenges in linking DNA methylation directly to protein abundance in multifactorial diseases like HIV.

Previous studies have linked specific HLA SNPs and alleles with HIV control,^18^^,^^69^ prompting us to investigate whether epigenetic modifications may mediate such an association. Our analysis indeed revealed a significant association between hypermethylation at cg17974398 and rs3131018-C near *POU5F1* in HIV controllers. Mediation analysis suggests that the effect of rs3131018-C may be mediated by hypermethylation of cg17974398, thereby contributing to HIV control. cg17974398 is located in an intron of *HLA-*C, an MHC I protein reported to play a crucial role by transmitting inhibitory signals to NK cells and cytotoxic T lymphocytes (CTL) through interaction with killer cell Ig-like receptors (KIR).^70^^,^^71^ *HLA-C* has also gained recognition as a pivotal molecule in the immune control of HIV-1. Furthermore, hypermethylation at cg07189782 and cg04784635-both inversely associated with key immune activation markers in CD4^+^ T cells (PD-1, HLA-DR, CD38, CCR5, and CXCR3)-may also contribute to viral control. However, despite this, our search of the GWAS Catalogue for HIV control–associated SNPs near *PRDX3* and *LHX5* yielded no significant findings, thereby limiting our ability to perform mediation analyses at these loci. Nevertheless, the potential causal role of methylation at cg07189782 and cg04784635 in HIV control remains a promising area for future investigation.

We also conducted an EWAS of HIV controllers in all ancestries. No DMS were replicated in the validation cohort. Further comparison between the 112 nominally validated DMSs from Western Europe suggested a very specific pattern between different ancestries, especially in the Asian population. This may suggest that the methylation pattern is largely population specific, however, due to the small sample size of the Asian population, this conclusion should be interpreted with caution. On the other hand, we did find that methylation patterns in the MHC region near *TNF,* and *LTB* are common to all ancestries. DNA methylation is also reported to exhibit significant divergence between populations, primarily attributable to variations in allele frequencies and intricate interactions between genes and the environment.^72^^,^^73^ In summary, these findings emphasise the importance of hypermethylation and the MHC region in HIV control. Also, the significance of identifying population or ancestry-specific HIV controller signatures for the development of therapeutic targets to control HIV. Additionally, although universal initiation of ART is now the global standard of care regardless of viral load or CD4 count, our study underscores that elite controllers and viraemic controllers remain a valuable model for investigating the mechanisms of durable viral suppression and immune regulation in the absence of therapy. Rather than proposing an alternative to ART, this work aims to expand understanding of host–virus interactions, immune pathways, and epigenetic regulation, which may ultimately inform the development of future therapeutic strategies, including those directed toward achieving a functional cure or sustained ART-free remission.

Despite this being the largest EWAS of HIV control, this study has several limitations. The validation cohort was small (n = 12, HIV controllers) and did not include ECs. which limits statistical power and likely explains the low replication rate (3/597 CpG sites) observed. Future scientific studies may be able to increase the sample size, especially of EC, to profile the DNA methylation landscape in this rare group of PWH. Although age and sex were significantly associated with DNA methylation variation, their effects were adjusted for in the discovery cohort. In the validation cohort, only age was corrected due to its stronger influence and to avoid overfitting given the limited sample size of the HIV control group, following the “1 in 10 rule.” Moreover, our study did not include longitudinal data of both persistent and transient controllers, which further limits the conclusions of having the identified DNA methylation profile long-term memory feature driving HIV control. In the near future, the potential causal role of methylation at cg07189782 and cg04784635-which are inversely associated with key immune markers (e.g., PD-1, HLA-DR, CD38, CCR5, and CXCR3) in CD4^+^ T cells-should also be explored to better understand their involvement in HIV pathogenesis and immune regulation. As this was a cross-sectional analysis, the study is limited to identifying associations and cannot establish causality. Although no mechanistic experiments were conducted, the significant findings may serve as a valuable resource for future investigations into the biological pathways underlying spontaneous HIV control. Since we profiled the DNA methylation in whole blood and the EPIC array only covers 3% of CpGs in the genome, future studies using bisulfite sequencing in a cell-type specific manner could be of interest to further unravel the mechanisms regulated through epigenetics in HIV controllers. In particular, profiling DNA methylation in sorted CD4^+^ T cells-which are directly involved in HIV pathogenesis-may provide more specific insights by minimising cellular heterogeneity and revealing regulatory signals that are masked in whole blood. Also, although our cohort of spontaneous controllers was enriched for HLA-B*57 and HLA-B*27 alleles, the extent to which these HLA types shape the DNA methylation landscape of HIV controller remains elusive and should be addressed in future studies. In the analysis of DMR, we could not validate CpG regions after correcting for multiple testing, and only results with nominal significance were shown. Lastly, the lack of post-treatment controllers, which restricts our ability to directly compare them with elite controllers and to explore potentially distinct underlying mechanisms of HIV control.

Collectively, our findings provide evidence that epigenetic mechanisms, particularly DNA methylation, play a significant role in HIV control. We identified methylation signatures in HIV controllers that regulate immune responses, particularly those dependent on MHC genes and inflammatory processes. However, the persistence of these epigenetic changes in transient controllers suggests that this mechanism is dispensable for the maintenance of spontaneous HIV control status and that other factors may also be involved, such as those involved in the modulation of inflammation-derived responses.

## Contributors

AvdV, MGN, JCdS, CJX and YL conceptualised and designed the study. MKG performed the DNA methylation data and integration analysis supervised by CJX. CJX, AvdV, MKG and JCdS wrote the manuscript. MJTB, LEvE, WAJWV, ALG and NV recruited the participants of the cohort and contributed to the acquisition and quality control of all datasets. AN analysed the flow cytometry dataset. LABJ, CR, AV, assisted during the establishment of the cohort. MKG, JCdS, VRV and SDER assembled the figures. XJ, ZL and OS validated EWAS results. JBB validated MR and mediation result. ZZ and VM performed imputation, and quality control of genotype data. AvdV and CJX supervised the study. MKG, XJ, ZL, OS and JBB accessed and verified the underlying data. All authors critically revised the manuscript for intellectual content. All authors read and approved the final version of the manuscript.

## Data sharing statement

DNA methylation, transcriptomics, cytokine and phenotype data have been deposited in the Radboud Data Repository (https://doi.org/10.34973/p96d-kz55). The plasma proteome data have been deposited at: https://data.ru.nl/collections/ru/rumc/2000hiv_r0004571_dsc_373. Code generated to process the data is freely available on Github (https://github.com/CiiM-Bioinformatics-group/2000HIV_HIV_controller).

## Declaration of interests

The authors are part of the 2000HIV study, which is supported by ViiV Healthcare. MGN is a scientific founder of TTxD, Biotrip, Lemba TX and Salvina TX. LABJ is a scientific founder of TTxD, Lemba TX and Salvina TX. JCdS, VM, VRV, SDER, and CJX received a *New Investigators Scholarship* to attend the Conference on Retroviruses and Opportunistic Infections (CROI) 2024 and 2025 to present data outside of the current manuscript. CR received investigator-initiated study support from Gilead and ViiV (aware.hiv) within the past 36 months, has served on advisory boards for Gilead, ViiV, and MSD, is a member of the EACS HIV Guideline Committee, and holds stock in Immunocore. The remaining authors declare no competing interests.

## Appendix A Supplementary data

Supplementary Tables

Extended Figure
