# Supplementary material for: DNA methylation landscapes of HIV controllers: an epigenome-wide association study
Source: eBioMedicine. 2025 Oct 30;121:105999. doi: 10.1016/j.ebiom.2025.105999 (PMC12598396; doi:10.1016/j.ebiom.2025.105999)
Supplement: Extended Figure [file mmc2.pptx]

## Slide 1
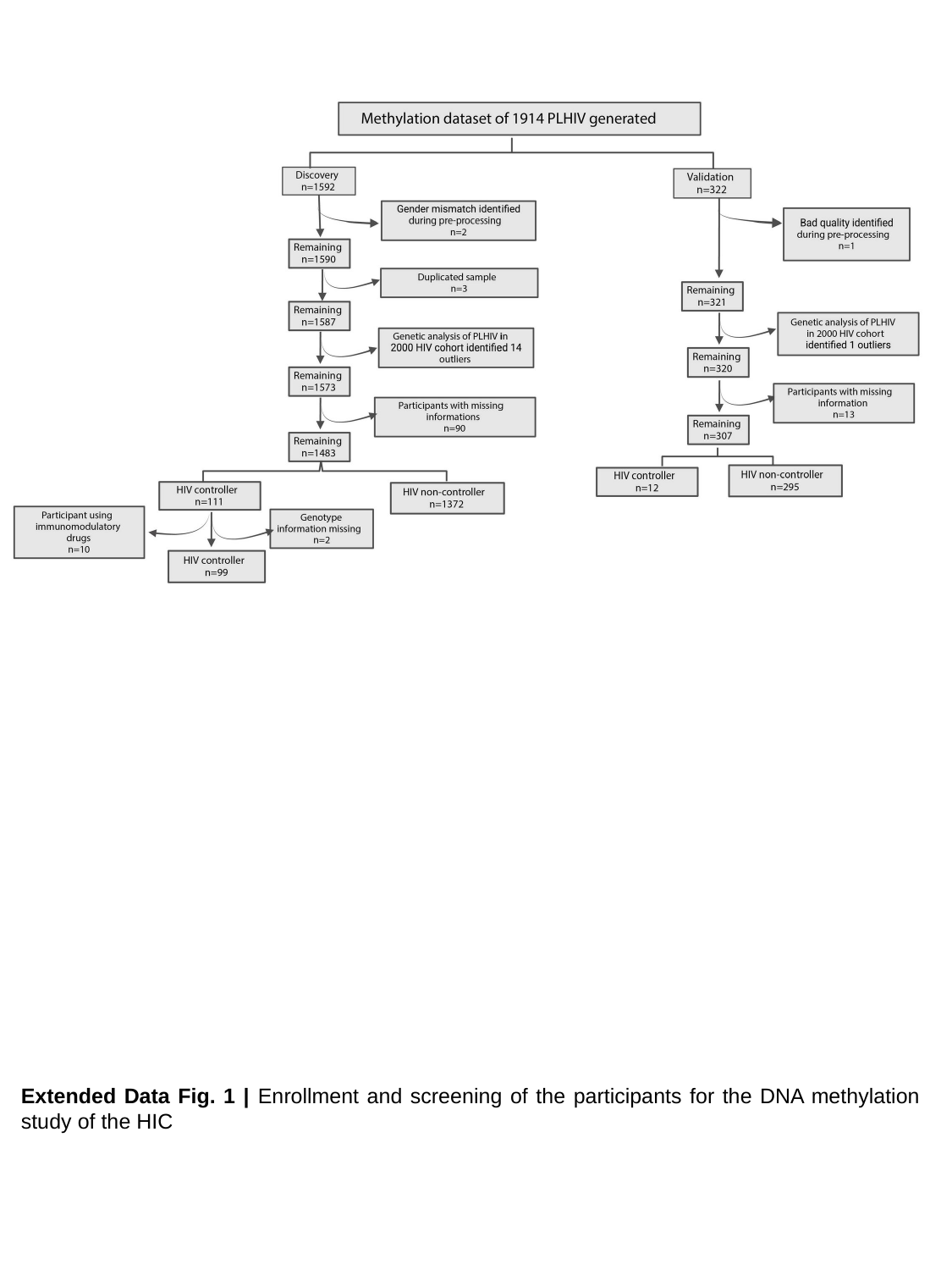

Extended Data Fig. 1 | Enrollment and screening of the participants for the DNA methylation study of the HIC

## Slide 2
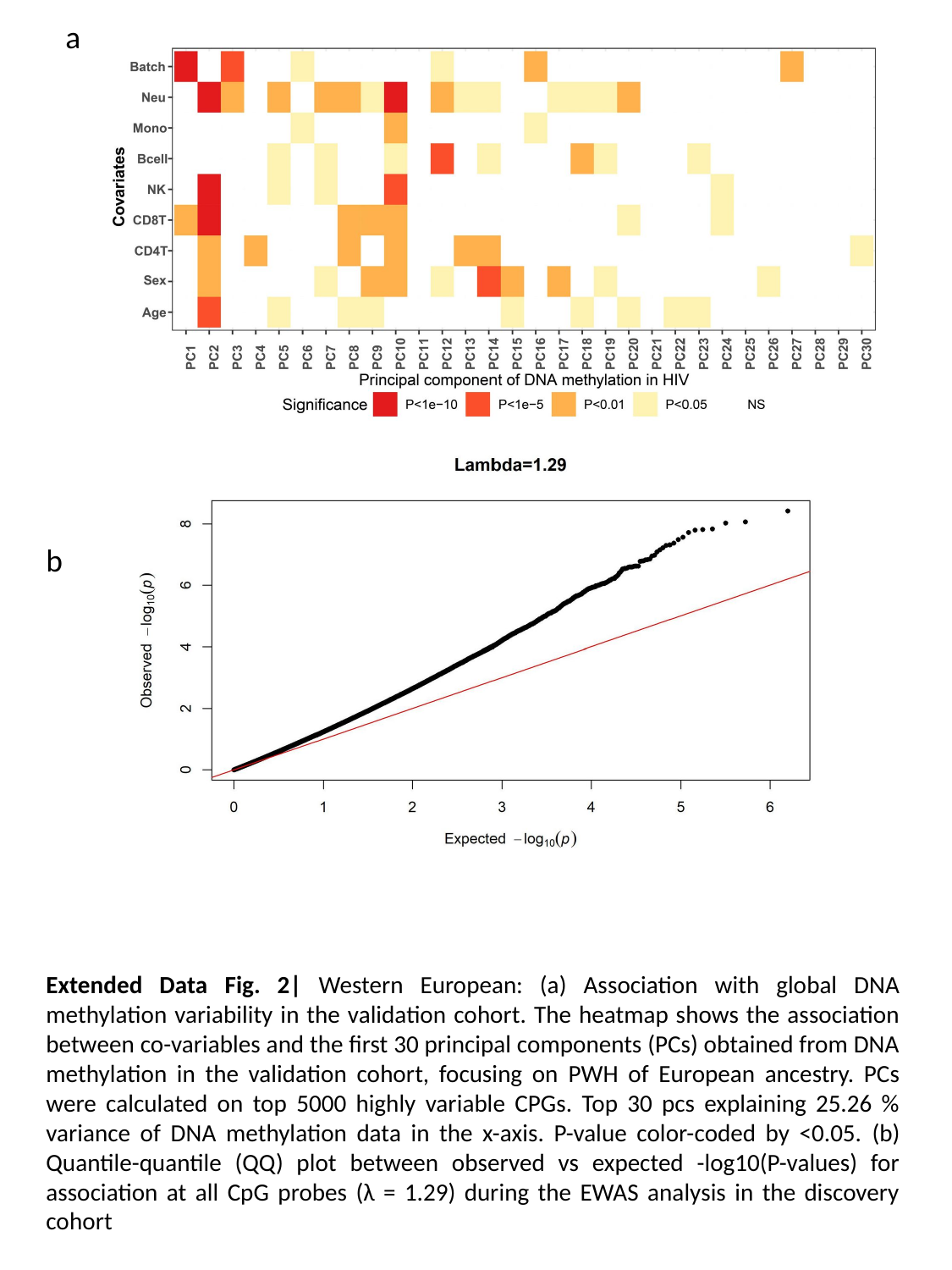

a
b
Extended Data Fig. 2| Western European: (a) Association with global DNA methylation variability in the validation cohort. The heatmap shows the association between co-variables and the first 30 principal components (PCs) obtained from DNA methylation in the validation cohort, focusing on PWH of European ancestry. PCs were calculated on top 5000 highly variable CPGs. Top 30 pcs explaining 25.26 % variance of DNA methylation data in the x-axis. P-value color-coded by <0.05. (b) Quantile-quantile (QQ) plot between observed vs expected -log10(P-values) for association at all CpG probes (λ = 1.29) during the EWAS analysis in the discovery cohort

## Slide 3
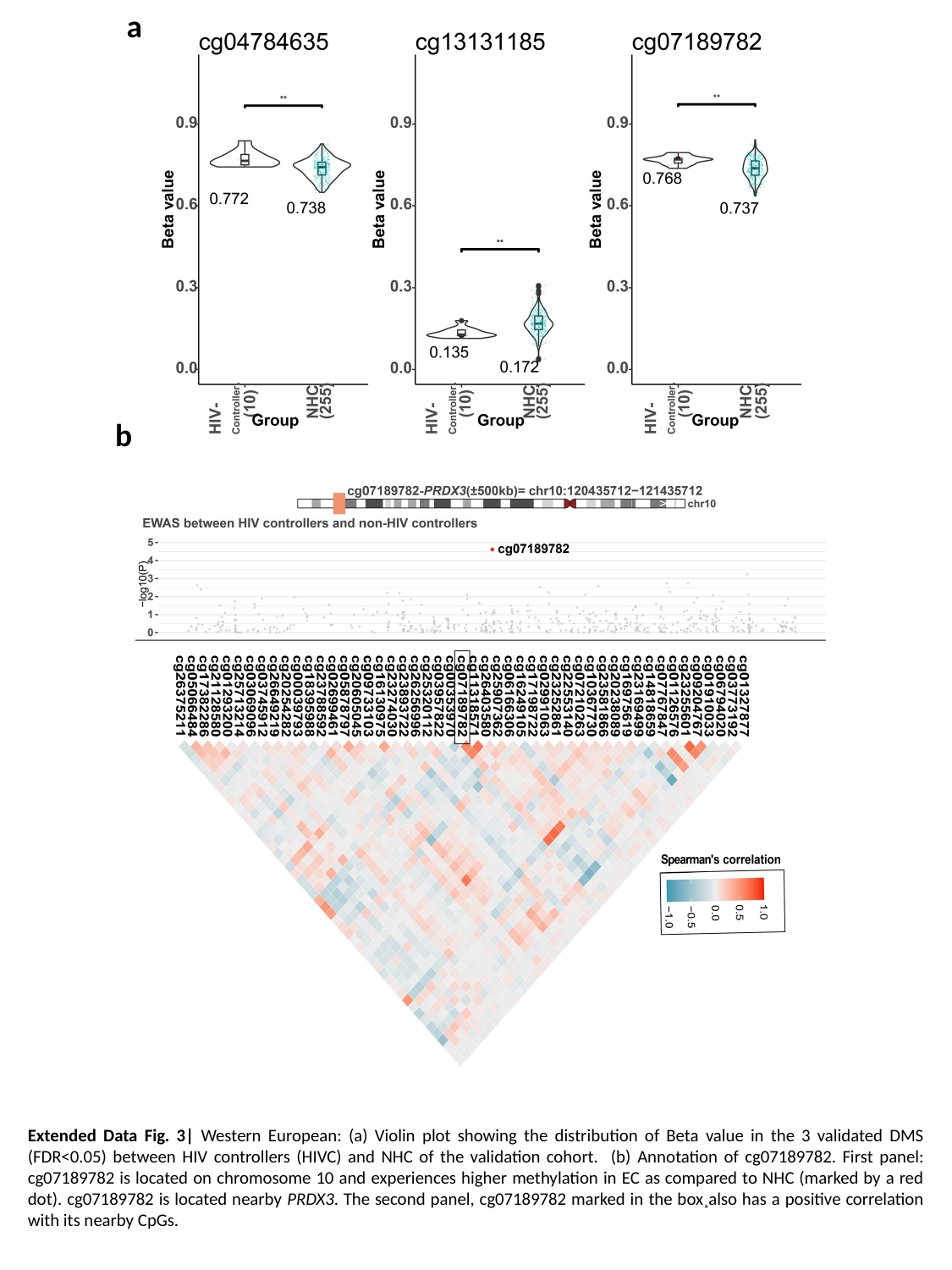

a
b
Extended Data Fig. 3| Western European: (a) Violin plot showing the distribution of Beta value in the 3 validated DMS (FDR<0.05) between HIV controllers (HIVC) and NHC of the validation cohort. (b) Annotation of cg07189782. First panel: cg07189782 is located on chromosome 10 and experiences higher methylation in EC as compared to NHC (marked by a red dot). cg07189782 is located nearby PRDX3. The second panel, cg07189782 marked in the box¸also has a positive correlation with its nearby CpGs.

## Slide 4
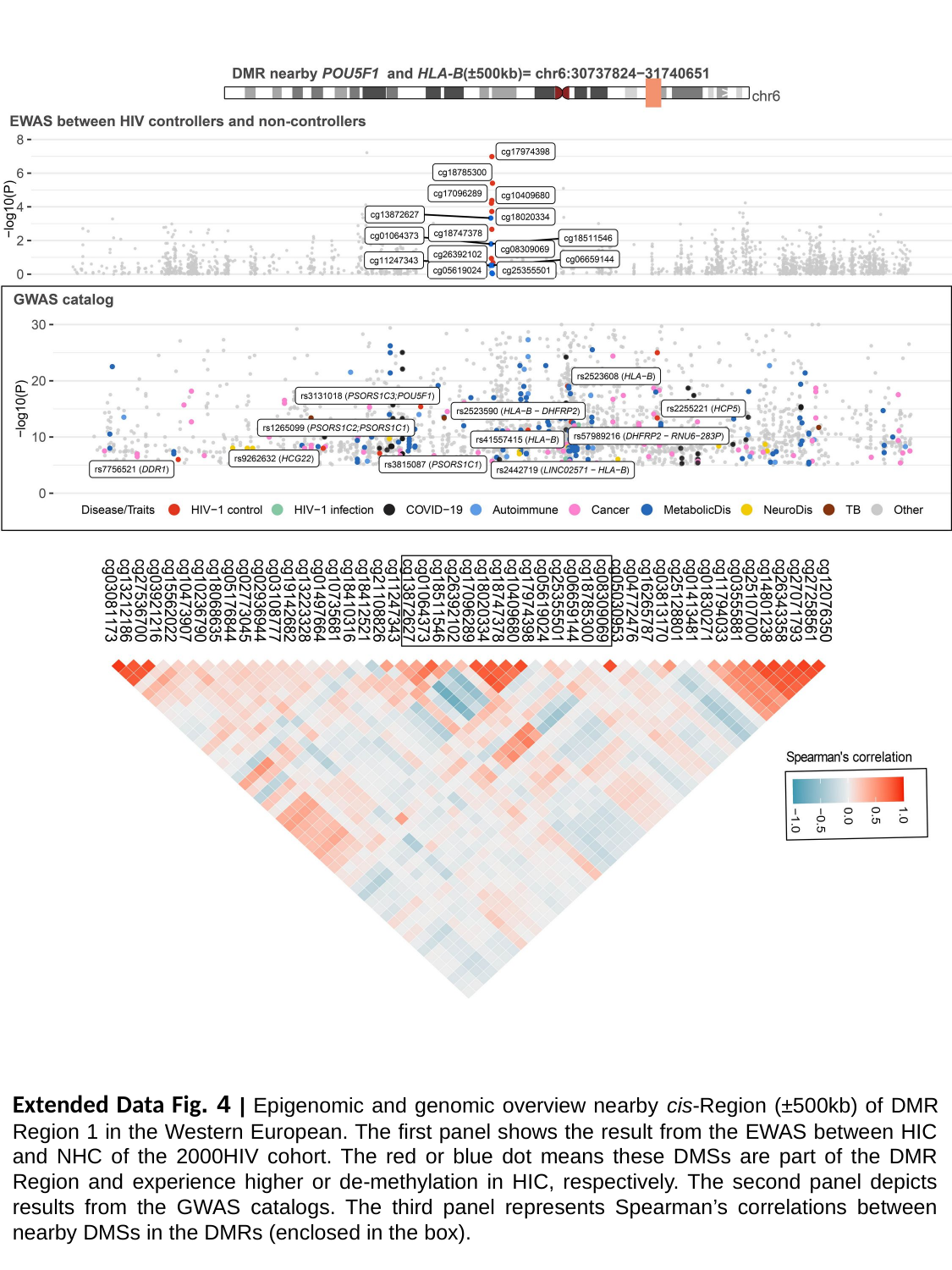

Extended Data Fig. 4 | Epigenomic and genomic overview nearby cis-Region (±500kb) of DMR Region 1 in the Western European. The first panel shows the result from the EWAS between HIC and NHC of the 2000HIV cohort. The red or blue dot means these DMSs are part of the DMR Region and experience higher or de-methylation in HIC, respectively. The second panel depicts results from the GWAS catalogs. The third panel represents Spearman’s correlations between nearby DMSs in the DMRs (enclosed in the box).

## Slide 5
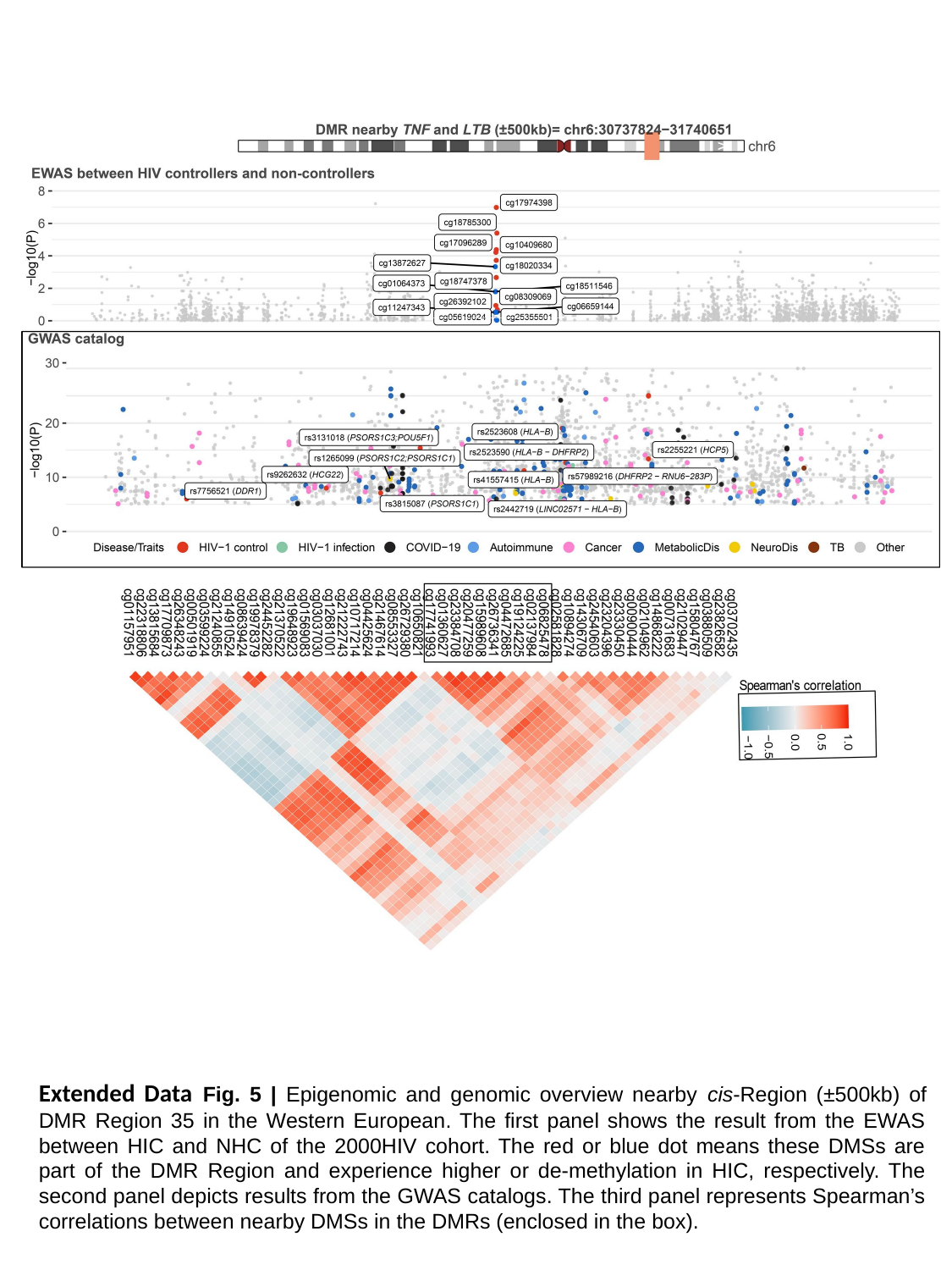

Extended Data Fig. 5 | Epigenomic and genomic overview nearby cis-Region (±500kb) of DMR Region 35 in the Western European. The first panel shows the result from the EWAS between HIC and NHC of the 2000HIV cohort. The red or blue dot means these DMSs are part of the DMR Region and experience higher or de-methylation in HIC, respectively. The second panel depicts results from the GWAS catalogs. The third panel represents Spearman’s correlations between nearby DMSs in the DMRs (enclosed in the box).

## Slide 6
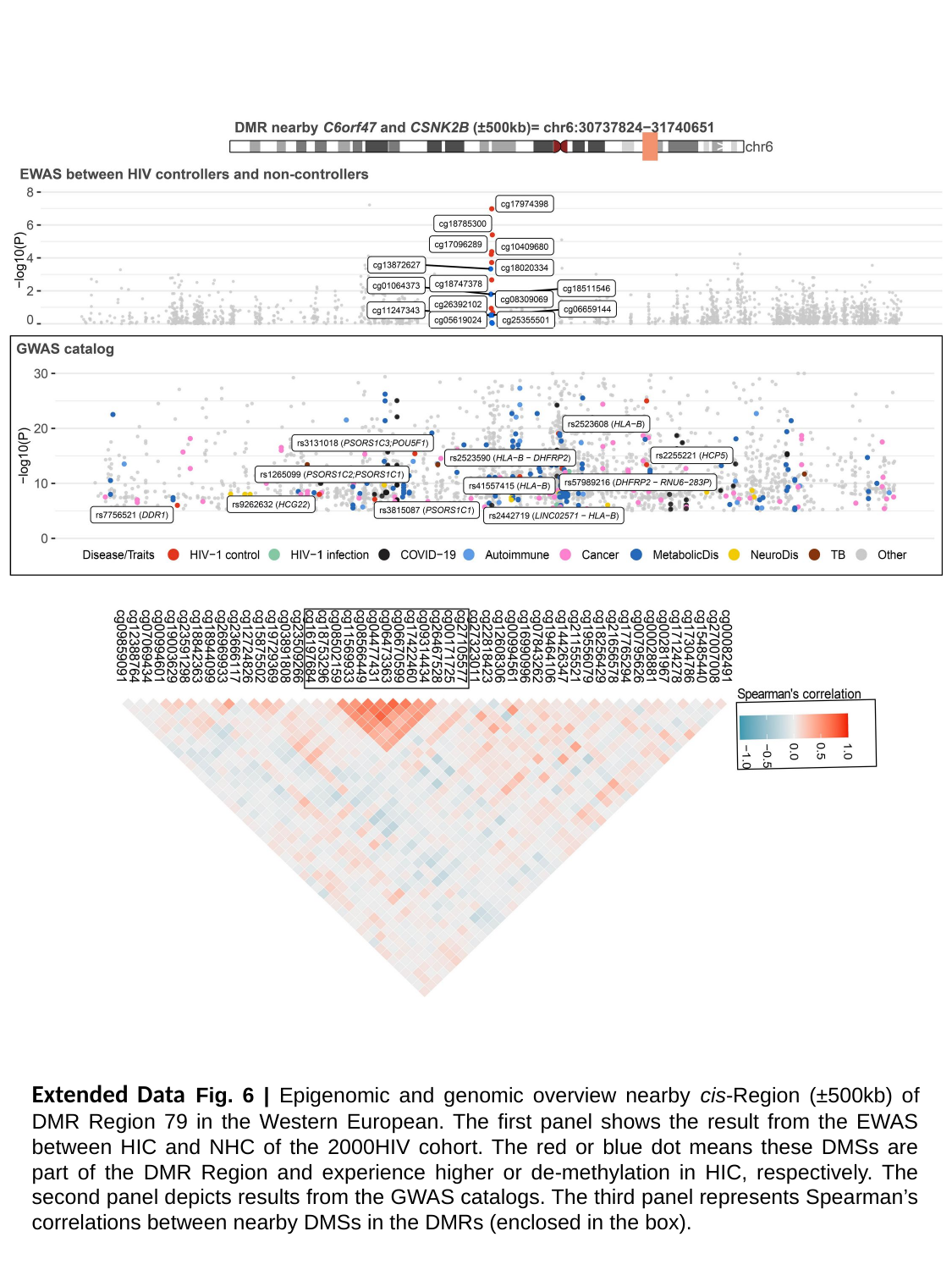

Extended Data Fig. 6 | Epigenomic and genomic overview nearby cis-Region (±500kb) of DMR Region 79 in the Western European. The first panel shows the result from the EWAS between HIC and NHC of the 2000HIV cohort. The red or blue dot means these DMSs are part of the DMR Region and experience higher or de-methylation in HIC, respectively. The second panel depicts results from the GWAS catalogs. The third panel represents Spearman’s correlations between nearby DMSs in the DMRs (enclosed in the box).

## Slide 7
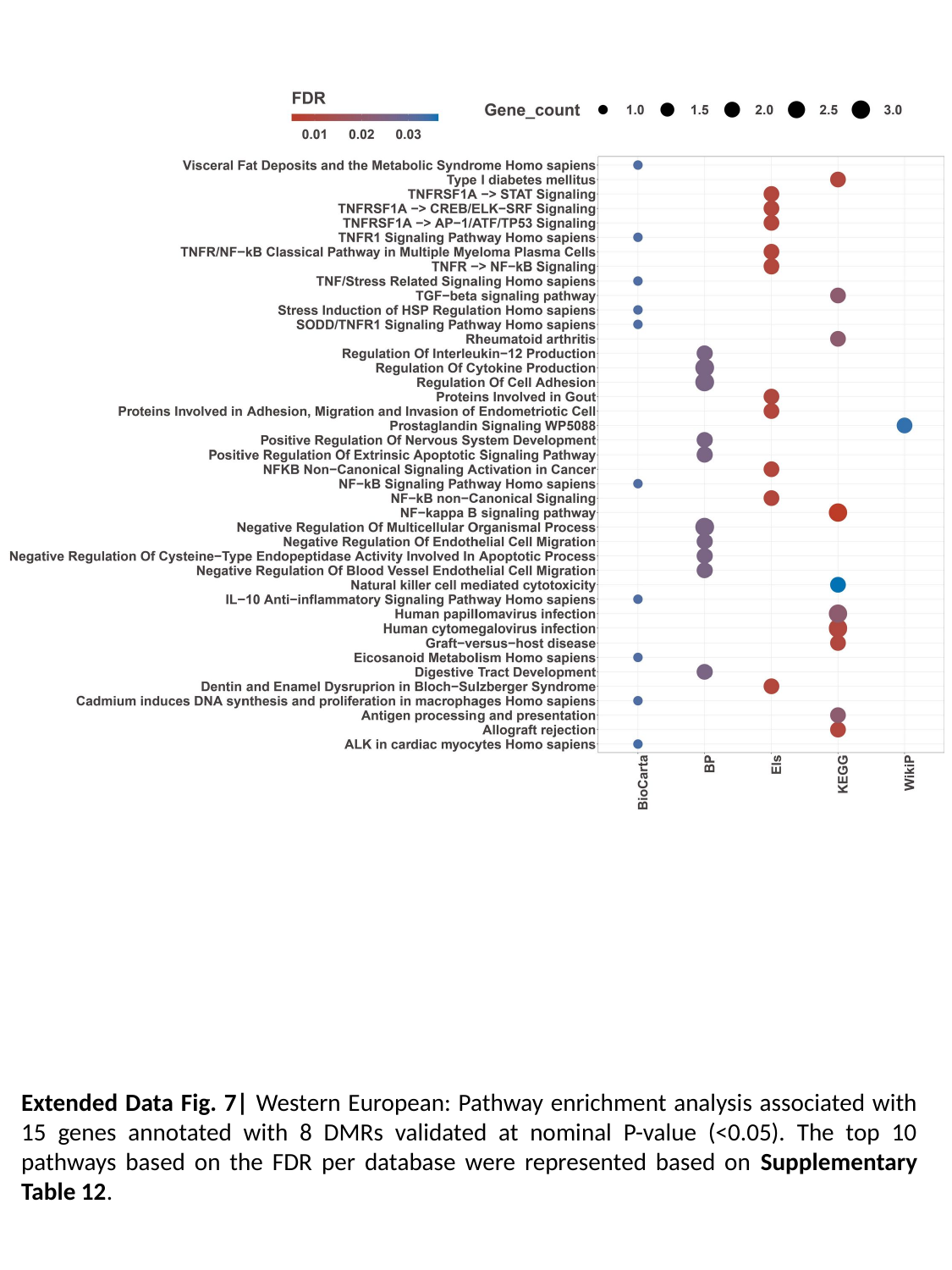

Extended Data Fig. 7| Western European: Pathway enrichment analysis associated with 15 genes annotated with 8 DMRs validated at nominal P-value (<0.05). The top 10 pathways based on the FDR per database were represented based on Supplementary Table 12.

## Slide 8
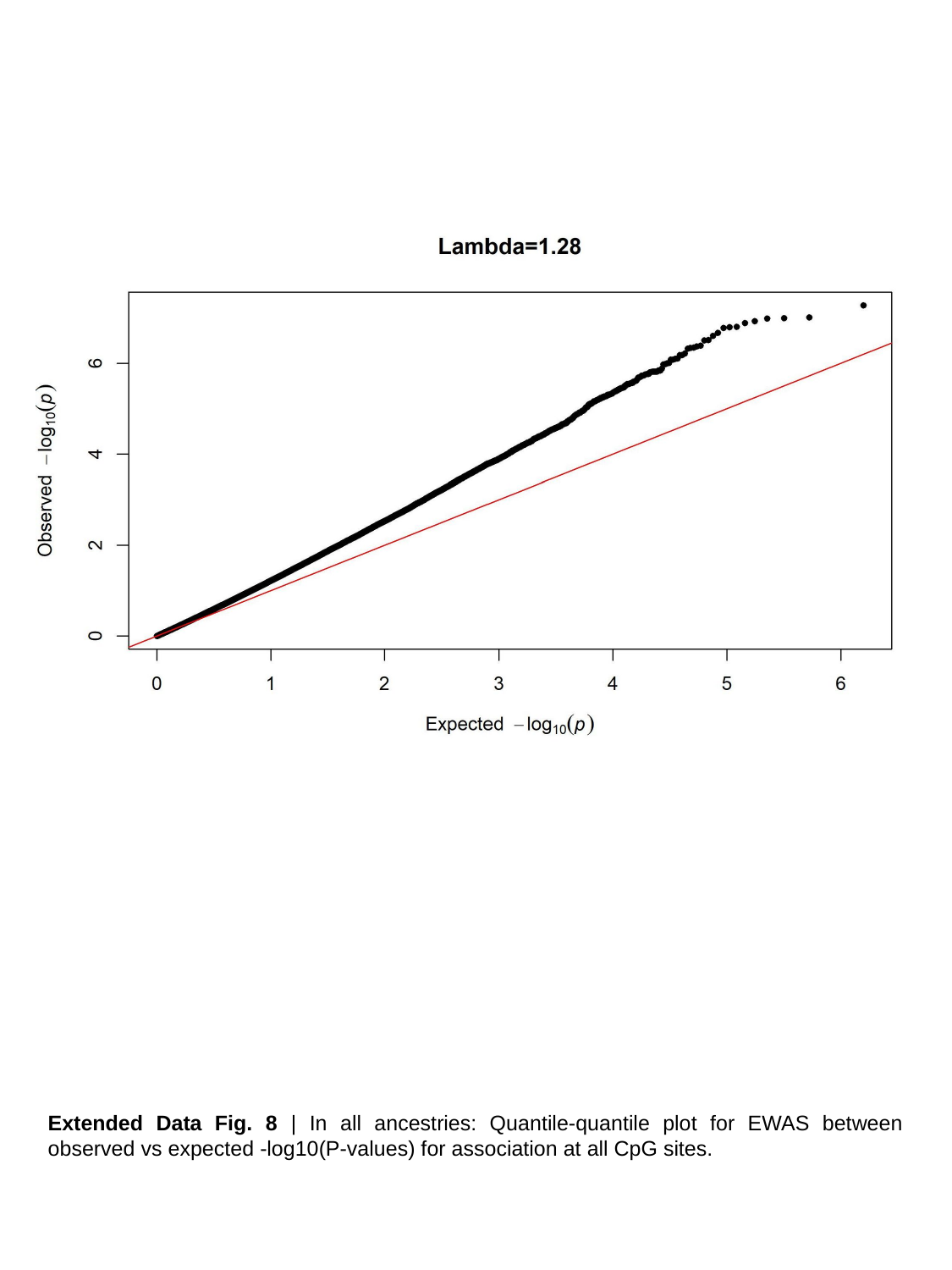

Extended Data Fig. 8 | In all ancestries: Quantile-quantile plot for EWAS between observed vs expected -log10(P-values) for association at all CpG sites.

## Slide 9
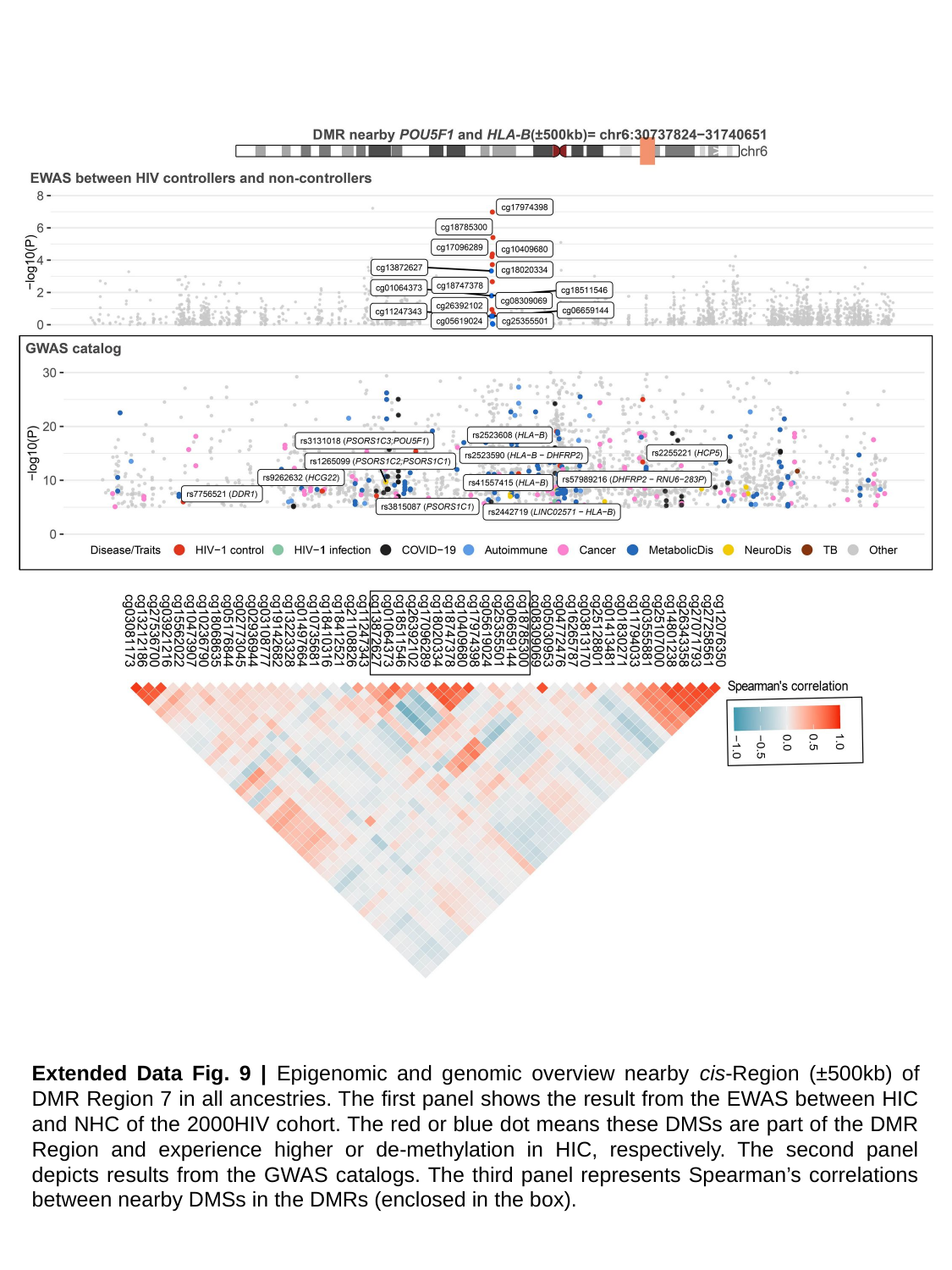

Extended Data Fig. 9 | Epigenomic and genomic overview nearby cis-Region (±500kb) of DMR Region 7 in all ancestries. The first panel shows the result from the EWAS between HIC and NHC of the 2000HIV cohort. The red or blue dot means these DMSs are part of the DMR Region and experience higher or de-methylation in HIC, respectively. The second panel depicts results from the GWAS catalogs. The third panel represents Spearman’s correlations between nearby DMSs in the DMRs (enclosed in the box).

## Slide 10
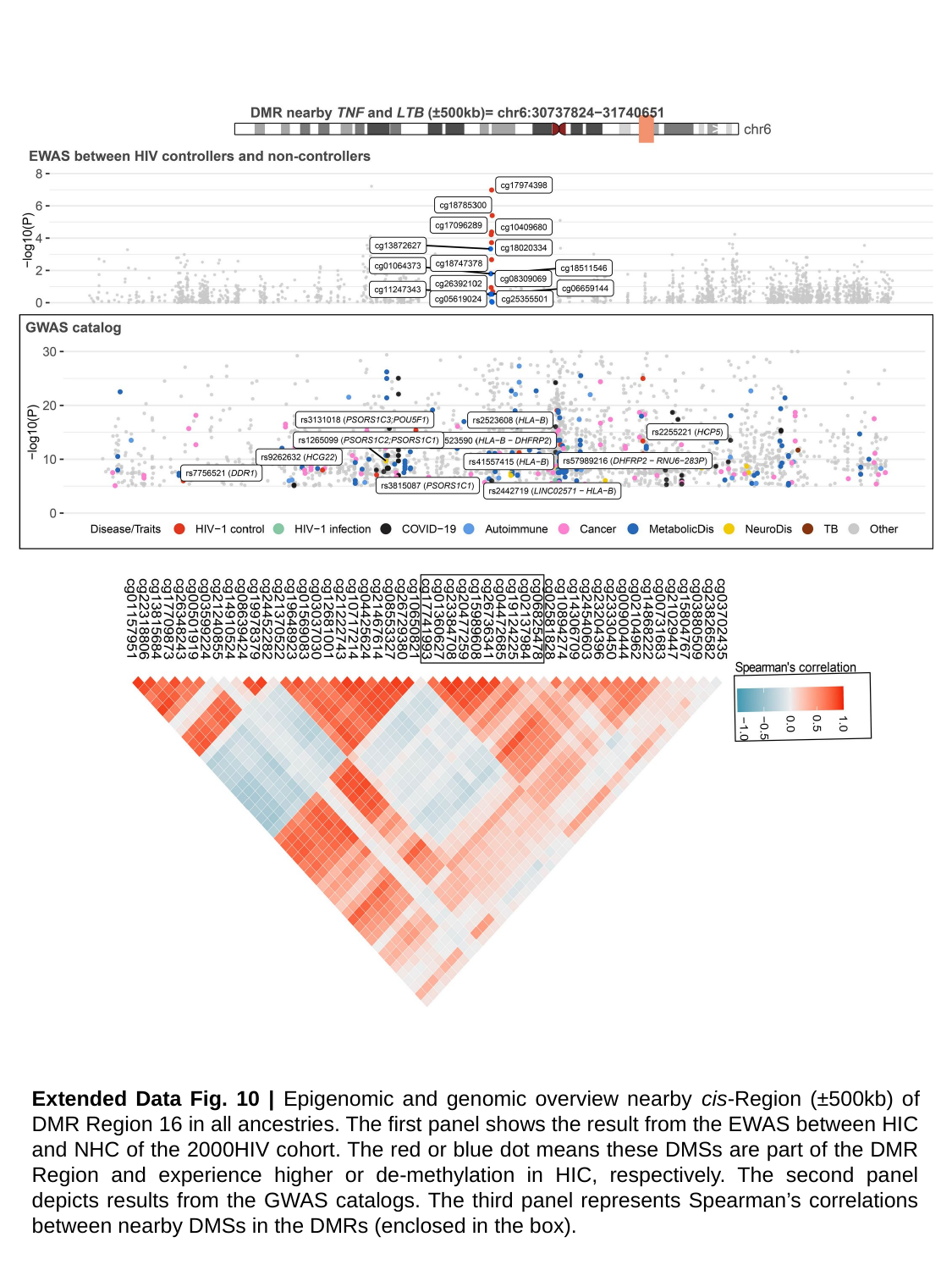

Extended Data Fig. 10 | Epigenomic and genomic overview nearby cis-Region (±500kb) of DMR Region 16 in all ancestries. The first panel shows the result from the EWAS between HIC and NHC of the 2000HIV cohort. The red or blue dot means these DMSs are part of the DMR Region and experience higher or de-methylation in HIC, respectively. The second panel depicts results from the GWAS catalogs. The third panel represents Spearman’s correlations between nearby DMSs in the DMRs (enclosed in the box).
